# Advances in research on immunocyte iron metabolism, ferroptosis, and their regulatory roles in autoimmune and autoinflammatory diseases

**DOI:** 10.1038/s41419-024-06807-2

**Published:** 2024-07-04

**Authors:** Liuting Zeng, Kailin Yang, Ganpeng Yu, Wensa Hao, Xiaofei Zhu, Anqi Ge, Junpeng Chen, Lingyun Sun

**Affiliations:** 1 Department of Rheumatology and Immunology, Nanjing Drum Tower Hospital, Chinese Academy of Medical Sciences and Peking Union Medical College, Graduate School of Peking Union Medical College, Nanjing, China; 2 Key Laboratory of Hunan Province for Integrated Traditional Chinese and Western Medicine on Prevention and Treatment of Cardio-Cerebral Diseases, School of Integrated Chinese and Western Medicine, Hunan University of Chinese Medicine, Changsha, China; 3 Psychosomatic laboratory, Department of Psychiatry, Daqing Hospital of Traditional Chinese Medicine, Daqing, China; 4 People’s Hospital of Ningxiang City, Ningxiang, China; 5 Institute of Materia Medica, Chinese Academy of Medical Sciences & Peking Union Medical College, Beijing, China; 6 Fudan University, Shanghai, China; 7 The First Hospital of Hunan University of Chinese Medicine, Changsha, Hunan China; 8 Department of Physiology, School of Medicine, University of Louisville, Louisville, KY USA; 9 College of Mechanical Engineering, Hunan University of Science and Technology, Xiangtan, China; 10 Department of Rheumatology and Immunology, The First Affiliated Hospital of Anhui Medical University, Hefei, China

**Keywords:** Immunology, Immunological disorders

## Abstract

Autoimmune diseases commonly affect various systems, but their etiology and pathogenesis remain unclear. Currently, increasing research has highlighted the role of ferroptosis in immune regulation, with immune cells being a crucial component of the body’s immune system. This review provides an overview and discusses the relationship between ferroptosis, programmed cell death in immune cells, and autoimmune diseases. Additionally, it summarizes the role of various key targets of ferroptosis, such as GPX4 and TFR, in immune cell immune responses. Furthermore, the release of multiple molecules, including damage-associated molecular patterns (DAMPs), following cell death by ferroptosis, is examined, as these molecules further influence the differentiation and function of immune cells, thereby affecting the occurrence and progression of autoimmune diseases. Moreover, immune cells secrete immune factors or their metabolites, which also impact the occurrence of ferroptosis in target organs and tissues involved in autoimmune diseases. Iron chelators, chloroquine and its derivatives, antioxidants, chloroquine derivatives, and calreticulin have been demonstrated to be effective in animal studies for certain autoimmune diseases, exerting anti-inflammatory and immunomodulatory effects. Finally, a brief summary and future perspectives on the research of autoimmune diseases are provided, aiming to guide disease treatment strategies.

## Facts

- Ferroptosis is a recently defined form of programmed cell death that uniquely links elements such as iron, selenium, amino acids, lipids, and redox chemistry in cell metabolism into a cohesive network.
- Both in vitro and in vivo experiments support the regulation of ferroptosis in various immune cells through multiple metabolic signaling pathways, including innate and adaptive pathways.
- Ferroptosis is involved in the pathophysiological processes of systemic organ dysfunction and tissue damage in autoimmune and inflammatory diseases.
- Therapeutic targeting of the ferroptotic pathway may provide new treatment opportunities for autoimmune and inflammatory diseases that were previously untreatable or had treatment failures and relapses.

## Open questions

- How much do we know about the involvement of ferroptosis in innate and adaptive immune pathways?
- How should we explore the molecular mechanisms of ferroptosis in autoimmune and inflammatory diseases?
- Does targeting the ferroptotic pathway offer new opportunities for drug development in future autoimmune and inflammatory diseases?
- Does targeting the ferroptotic pathway provide new treatment opportunities in the future for previously untreatable or treatment-resistant recurrent autoimmune and inflammatory diseases?

## Introduction

Autoimmune diseases are inflammatory disorders in which the body’s immune system erroneously attacks normal cells, leading to a reduction in normal immune function and an increase in abnormal immune responses, ultimately resulting in tissue damage or organ dysfunction [1]. Epidemiological studies [2] have demonstrated that around 10% of the global population is afflicted by autoimmune diseases, positioning them as the third most prevalent category of chronic illnesses following cardiovascular diseases and cancer. Autoimmune diseases are characterized by high disability rates, elevated mortality rates, and a significant impact on quality of life. Presently, there are over 80 autoimmune diseases without precise scientific definitions, along with conditions exhibiting autoimmune-related symptoms that share genetic and immunological mechanisms across various autoimmune disorders [3, 4]. Despite an incomplete understanding of autoimmune disease pathogenesis, these conditions exhibit several common characteristics: (1) often unclear etiology, with some cases being spontaneous or idiopathic, while others may be linked to bacterial or viral infections or certain medications; (2) higher prevalence in females compared to males; (3) a chronic and relapsing nature; (4) a distinct familial tendency; and (5) co-occurrence of multiple autoimmune diseases in a single patient [5, 6]. The pathogenesis of these illnesses involves the immune system mistakenly targeting healthy cells and tissues within the body, resulting in persistent inflammation, tissue damage, and organ dysfunction [7]. Throughout the pathological progression of autoimmune diseases, programmed cell death mechanisms such as autophagy, apoptosis, and pyroptosis predominantly contribute to chronic inflammation, tissue damage, and organ dysfunction [8–11]. Recent investigations [12] have introduced a newly identified mode of cell death termed ferroptosis, which plays a role in chronic inflammation and organ damage in autoimmune diseases. With a growing body of evidence supporting the involvement of ferroptosis in autoimmune diseases, it is apparent that this cellular demise pathway is ubiquitously present and pivotal in the pathogenesis of autoimmune disorders [13, 14].

**Fig. 1:**
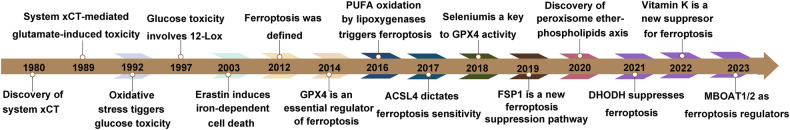
The key time points for the discovery of ferroptosis. From 1980 to the present, many studies on ferroptosis have been conducted. GPX4 glutathione peroxidase 4, PUFA polyunsaturated fatty acid.

Ferroptosis is a newly characterized iron-dependent form of programmed cell death characterized by elevated levels of reactive oxygen species (ROS), setting it apart from other programmed cell death modalities such as apoptosis, necrosis, and autophagy [15]. The primary mechanism underlying ferroptosis involves the catalytic action of divalent iron or lipoxygenases on highly expressed unsaturated fatty acids present on the cell membrane, provoking lipid peroxidation and ultimately triggering cell demise. Consequently, cellular iron homeostasis, polyunsaturated fatty acid (PUFA) metabolism and oxidation, and antioxidant systems play crucial roles in influencing ferroptosis, involving a cascade of protein regulatory mechanisms. These encompass proteins like transferrin receptor (TFRC) governing iron homeostasis, solute carrier family 40 member 1 (SLC40A1) or ferroportin (FPN) implicated in iron transport, and ferritin responsible for iron storage. Additionally, proteins such as acetyl CoA carboxylase (ACC), acyl-CoA synthetase long-chain family member 4 (ACSL4), lysophosphatidylcholine acyltransferase 3 (LPCAT3), and lipoxygenases (LOX) influence PUFA metabolism and oxidation, while glutathione peroxidase 4 (GPX4) fulfills a crucial role in the antioxidant system by directly converting lipid hydroperoxides to lipid alcohols [16, 17]. In terms of morphology, ferroptosis leads to smaller mitochondria characterized by increased membrane density and reduced cristae, while the nucleus remains intact [18]. Recent studies have highlighted the intimate link between iron metabolism, ferroptosis, and numerous human diseases, including cardiovascular diseases, tumorigenesis, neurodegenerative disorders, and ischemia-reperfusion injury [19–23]. Nevertheless, a comprehensive review and synthesis focusing specifically on autoimmune diseases are notably absent. This review seeks to comprehensively analyze and consolidate foundational research conducted by our team and other research cohorts, with the objective of elucidating the interplay between ferroptosis and various immune cells (such as macrophages, neutrophils, lymphocytes, etc.) as well as the parenchymal cells of target organs in autoimmune diseases, thereby shedding light on the intricate relationship between ferroptosis and immune cells [24–27]. Figure 1 illustrates the key milestones in the discovery of ferroptosis.

## Ferroptosis and its characteristics

Programmed cell death is a genetically controlled process of cellular demise that unfolds in an active and organized manner, intricately linked with the maintenance of organismal homeostasis and the onset of diseases. Diverse forms of programmed cell death encompass apoptosis, pyroptosis, necrosis, and autophagy [28, 29]. Ferroptosis, a recently unveiled subtype of programmed cell death, is characterized by iron-triggered oxidative damage to polyunsaturated fatty acids (PUFAs) [30]. Investigations have unveiled that ferroptosis uniquely propagates in a wave-like fashion across a cell population, giving rise to distinctive spatiotemporal patterns of cell demise not seen in other cell death modalities. Riegman et al. observed that ferroptosis induces the formation of plasma membrane pores, facilitating solute exchange with the extracellular milieu and prompting cellular swelling [14]. In this sequence, the cellular swelling marker cPLA2 relocates to the nuclear membrane. Subsequent to cellular rupture, the dye SYTOX Green swiftly permeates the cells. Induction of ferroptosis by erastin, C’ dot, or FAC and BSO leads to its dissemination to neighboring cells through an iron and lipid peroxide-dependent mechanism, whereas the GPX4 inhibitor ML162 fails to transmit the ferroptosis signal to adjacent cells. Moreover, the researchers unveiled that ferroptosis propagation entails calcium flux but does not necessitate cellular rupture, thus permitting the transmission of the ferroptosis signal to neighboring cells in an iron and lipid peroxide-dependent manner. Ferroptosis is intricately associated with a spectrum of human diseases, including cancer, aging, neurodegenerative disorders, and ischemia-reperfusion injury [31]. Notably, a study elucidated that the ferroptosis inducer sulfasalazine significantly impedes the management of rheumatic skeletal disorders [32].

### Morphological characteristics of ferroptosis

**Fig. 2:**
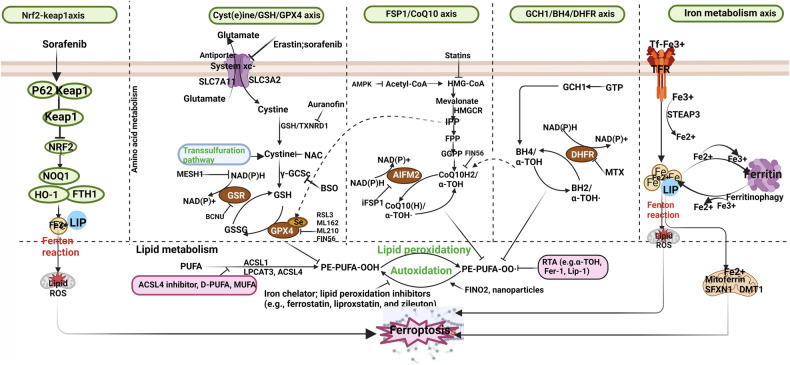
The molecular mechanism of ferroptosis. Those are the pathways involved in ferroptosis. Fer-1 ferrostatin-1, LIP labile Fe pool, NCOA4 nuclear receptor coactivator 4, DHFR dihydrofolate reductase, NRF2 nuclear factor-erythroid 2-related factor 2, ROS reactive oxygen species, GSR glutathione reductase, FPN ferroportin, FTH/FTL ferritin heavy chain/ ferritin light chain, TFR1 transferrin receptor 1, GPX4 glutathione peroxidase 4, FSP1 ferroptosis inhibitor protein 1, Lip-1 liproxstatin-1, (figures adapted from Zhang et al. [358], licensed under CC BY 4.0).

The morphological characteristics of ferroptosis primarily encompass disruptions in plasma membrane integrity, swelling of cytoplasm and organelles, and chromatin condensation. Moreover, notable alterations manifest in mitochondrial structure, including mitochondrial constriction, heightened membrane density, reduced or vanished cristae, and outer membrane rupture [33]. Additionally, ferroptosis is typified by cell detachment and aggregation, alongside an escalation in intracellular autophagic vesicles. These morphological attributes distinguish ferroptosis from cell death modalities like necrosis and apoptosis [33]. The molecular intricacies of ferroptosis are elucidated in Fig. 2.

### Biochemical characteristics of ferroptosis

Ferroptosis is a form of cell death closely associated with the accumulation of ROS, primarily characterized by iron accumulation and lipid peroxidation. When the cellular antioxidant system undergoes metabolic dysregulation, the accumulation of Fe2+ can mediate the Fenton reaction, resulting in the excessive generation of ROS, particularly hydroxyl radicals. These ROS react with PUFAs on the cell membrane, leading to lipid bilayer destabilization, cell membrane disruption, and ultimately promoting cell death via ferroptosis [34].

### Molecular mechanism of ferroptosis

As a form of programmed cell death, ferroptosis is closely linked to cellular amino acid, lipid, and iron metabolism [17]. Furthermore, other metabolic pathways and related factors can also influence the sensitivity of cells to ferroptosis.

#### Amino acid metabolism and ferroptosis

The system Xc- functions as a cystine-glutamate antiporter located on the cellular membrane surface, comprising the light chain SLC7A11 and the heavy chain SLC3A2, interconnected through a disulfide bond. It facilitates the reciprocal exchange of intracellular glutamate and extracellular cystine in a 1:1 ratio, ushering cystine entry into the cell. A reductive process transforms cystine into cysteine for glutathione (GSH) synthesis [35]. GPX4, a pivotal enzyme in the antioxidant defense system, employs GSH as a reducing agent to convert peroxides’ peroxyl bonds into hydroxyl groups, converting peroxides into alcohols and impeding ferroptosis. Ferroptosis activators include Erastin and Ras-selective lethal small molecule 3 (RSL3) [36]. Erastin binds to SLC7A11, hampering its function and disrupting cysteine transport, thus diminishing GSH production. Consequently, cells fail to effectively eliminate lipid peroxides, instigating membrane impairment and provoking ferroptosis [37]. Moreover, Erastin impacts voltage-dependent anion channels, disrupting cellular and organelle membranes (especially mitochondria), leading to the liberation of oxidative agents and culminating in cell demise [38]. RSL3, on the other hand, forms a covalent bond with GPX4, deactivating it and disturbing the cellular redox equilibrium, elevating lipid peroxidation and inducing ferroptosis [39]. Additionally, evidence suggests transcription factors regulate SLC7A11 expression to modulate ferroptosis. The nuclear transcription factor NEF2 serves as an inducer of SLC7A11 expression, while tumor suppressor genes TP53, BAP1, and BECN1 downregulate SLC7A11 expression [40–42]. Consequently, investigating agents targeting key components involved in GSH metabolism during ferroptosis holds substantial importance for comprehending and harnessing ferroptosis implications.

#### Lipid metabolism and ferroptosis

**Fig. 3:**
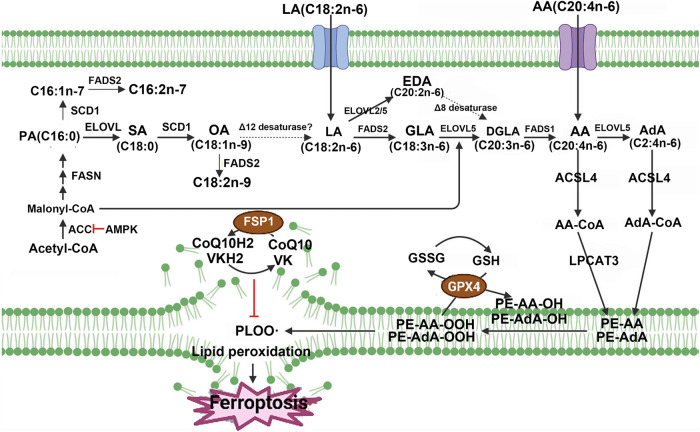
Fatty acid biosynthesis and ferroptosis. Fatty acid is involved in ferroptosis. AA arachidonic acid, CoA coenzyme A, PA palmitic acid, LA linoleic acid, (figures adapted from Kim et al. [359], licensed under CC BY 4.0).

**Fig. 4:**
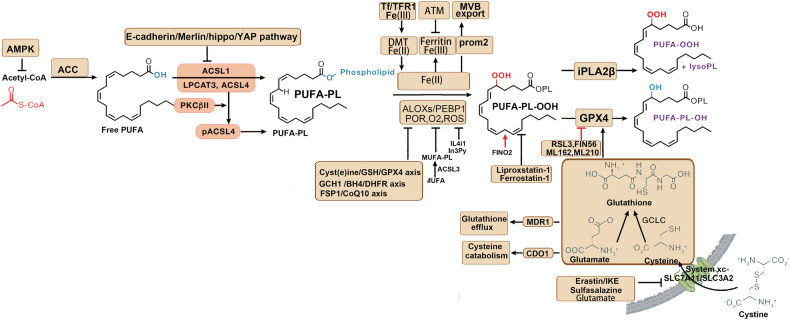
The mechanism by which lipid metabolism mediates ferroptosis. Lipid metabolism is involved in ferroptosis. AMPK adenosine-monophosphate-activated protein kinase, ACSL acyl-CoA synthetase long chain family member, CoQ10 coenzyme Q10, GPX4 glutathione peroxidase 4, GSH glutathione; iPLA2β phospholipase A2 group VI, PUFA polyunsaturated fatty acid.

PUFAs contain diene-propyl hydrogens, especially arachidonic acid (AA) and adrenic acid (AdA), which are prone to react with ROS, leading to lipid peroxidation and cell death via ferroptosis [43] (Fig. 3). Phosphatidylethanolamines (PEs) containing AA or AdA are critical phospholipids that induce ferroptosis. Long-chain ACSL4 catalyzes the binding of free AA or AdA with coenzyme A (CoA) to form derivatives AA-CoA or AdA-CoA, which are then esterified into membrane PEs by LPCAT3 [44]. Therefore, both increasing the sensitivity to ferroptosis by supplementing AA or other PUFAs and inhibiting the activity of ACSL4 and LPCAT3 can suppress ferroptosis [45]. The generation of ferroptosis signals requires the formation of CoA derivatives of PUFAs and their binding to phospholipids, which represents a potential target for treating diseases associated with ferroptosis. LOXs, a family of iron-containing proteins, can catalyze the peroxidation of PUFAs on cell membranes, inducing cell death via ferroptosis. In p53-induced ferroptosis, the p53 protein activates arachidonate-12-lipoxygenase (ALOX12), thus inducing cell death via ferroptosis, independent of GPX4 activity [46]. Inhibiting or downregulating ALOX12 may offer a novel approach to block ferroptosis [47]. The mechanism by which lipid metabolism mediates ferroptosis is shown in Fig. 4.

#### Iron metabolism and ferroptosis

**Fig. 5:**
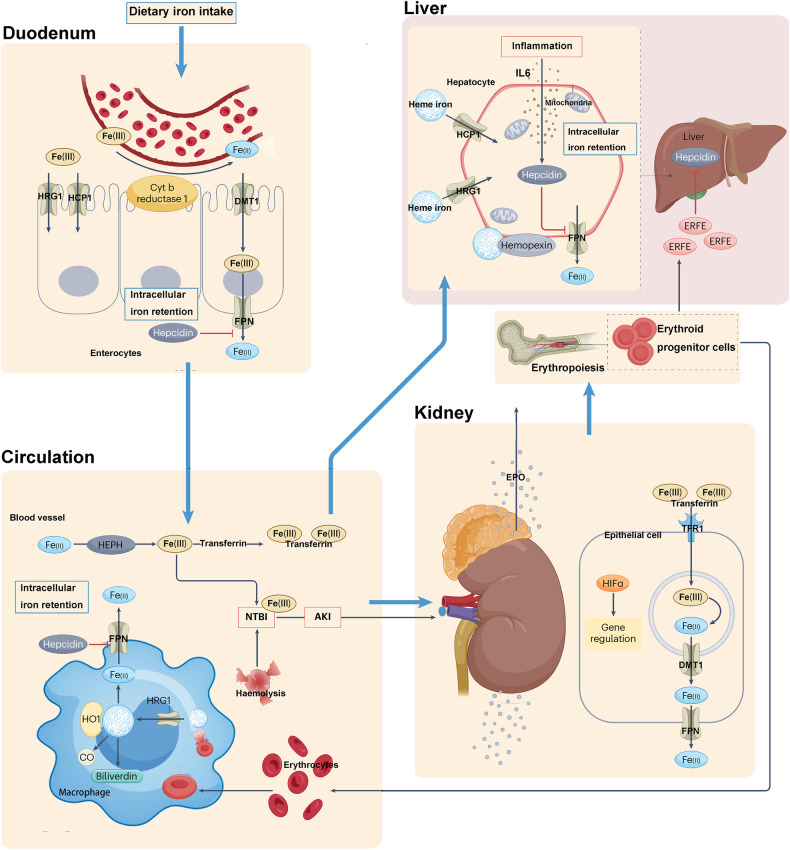
The metabolism of iron within the body. Iron metabolism is involved in many processes in the body. IL6 interleukin-6, TFR1 transferrin receptor 1.

Less than 1% of the total body iron is present in the extracellular space, while the majority of iron within mammalian cells (> 90%) exists as a co-factor for heme and iron-sulfur clusters, as well as single and dual iron centers in enzymes [48]. In addition to the small intestine, the kidney, liver, and macrophages play significant roles in maintaining systemic iron balance. Iron filtered by the renal glomerulus is actively reabsorbed to prevent urinary loss. The level of iron in the plasma is regulated by hepcidin, primarily synthesized by hepatocytes. However, other cells such as macrophages and the epithelial cells of the distal renal tubules also contribute to its synthesis to a lesser extent [48]. The metabolism of iron within the body is illustrated in Fig. 5. Iron exists in two oxidation states, Fe2+ and Fe3^+^. In the intestine, dietary Fe3+ is reduced to Fe2+ and subsequently absorbed by intestinal epithelial cells [48]. Under the action of membrane iron transport proteins, Fe2+ is transported out of the cell and oxidized to Fe3+ by multicopper oxidases. It then forms a complex with transferrin (TF) known as TF-Fe3+, which circulates through the bloodstream, delivering iron to various organs and tissues [49]. Fe3+ binds to transferrin receptors on the cell membrane and enters the cell. Once inside the cell, the intracellular metal reductase STEAP3 reduces Fe3+ back to Fe2 +, which is then released into the cytosolic labile iron pool by the divalent metal transporter 1 [49]. Under normal physiological conditions, the labile iron pool maintains iron balance. However, under pathological conditions, Fe2+ accumulates within the cell, engaging in the Haber-Weiss and Fenton reactions, leading to the generation of excessive ROS. ROS react with polyunsaturated fatty acids on the cell membrane, causing lipid peroxidation and ultimately disrupting the cell membrane structure, resulting in ferroptosis [50]. On the other hand, Fe2+ functions as a cofactor for various metabolic enzymes, enhancing the activity of enzymes such as LOXs and PDH1, thereby promoting ROS generation [51]. Therefore, factors related to iron metabolism represent potential targets for inducing ferroptosis. The tumor suppressor OTUD1 can bind to and promote the deubiquitination modification of iron response element binding protein 2 (IREB2), the main regulatory factor of iron metabolism. This stabilizes the IREB2 protein and subsequently activates the downstream TFRC expression. As a result, intracellular Fe2+ accumulates, ROS levels increase, and cellular sensitivity to ferroptosis is enhanced. Knocking down IREB2 can reduce cellular sensitivity to ferroptosis [52, 53]. Moreover, inhibiting TF activity has been found to decrease the occurrence of Fenton reactions, reducing ROS accumulation and lipid peroxidation, thereby inhibiting ferroptosis [52]. Deferoxamine can inhibit iron-induced ferroptosis, and limiting iron intake can alleviate liver damage caused by ferroptosis [54]. However, the precise mechanisms through which iron metabolism regulates ferroptosis remain unclear and require further exploration.

#### Other metabolic pathways and ferroptosis

**Fig. 6:**
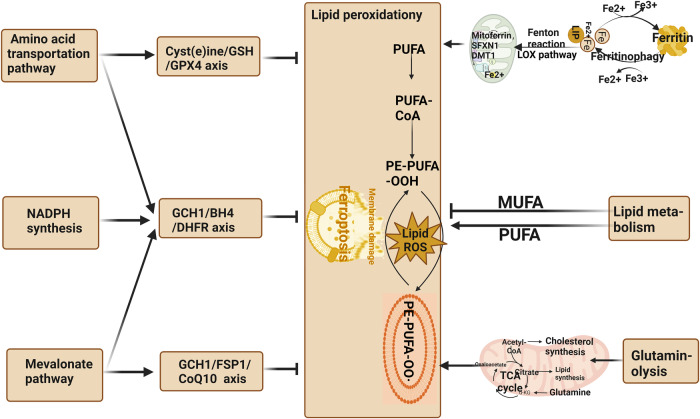
The pathways affecting the metabolic basis of ferroptosis. Multiple metabolic pathways are also involved in ferroptosis. MUFA monounsaturated fatty acid, PUFA polyunsaturated fatty acid, GPX4 glutathione peroxidase 4, GSH glutathione, (figures cited from Yuan et al. [360], licensed under CC BY-NC-ND 4.0).

Other factors that influence the sensitivity to ferroptosis include coenzyme Q10 (CoQ10), nicotinamide adenine dinucleotide phosphate (NADPH), selenium, p53, nuclear factor E2-related factor 2 (NRF2), nuclear factor erythroid 2-related factor 2 (NFE2L2), and Vitamin E. CoQ10 can be reduced by ferroptosis suppressor protein 1 (FSP1) to prevent lipid oxidation and inhibit ferroptosis [55]. Thus, FSP1 may serve as an important target for the treatment of related diseases. NADPH is involved in the cycling of the GPX4 antioxidant system, and its depletion limits the antioxidant function of GSH-GPX4, inducing ferroptosis [56, 57]. Selenium is an essential micronutrient that maintains GPX4 activity. It regulates the abundance and activity of GPX4 by coactivating transcription factors TFAP2c and Sp1, thereby inhibiting ferroptosis and protecting neurons [58]. The NFE2L2 signaling pathway represents an important defense mechanism against ferroptosis. NFE2L2 can transcriptionally activate relevant genes to limit oxidative damage during ferroptosis. These NFE2L2-regulated genes mainly include those involved in iron metabolism, GSH metabolism, and anti-ROS processes [59]. Vitamin E, as an endogenous antioxidant, protects cell membranes from oxidative damage. Studies have shown that GPX4 and Vitamin E work synergistically to protect cells, as Vitamin E inhibits ALOXs activity and reduces lipid peroxidation, thus inhibiting ferroptosis [60]. Therefore, Vitamin E can be a potential target for the treatment of ferroptosis. Additionally, transcription factors p53 and NRF2 also play significant roles in ferroptosis. Although various cell death pathways mediated by p53 have been studied for over 20 years, its involvement in ferroptosis has only recently been reported [61]. p53 can downregulate the expression of the Xc system component SLC7A11, inhibiting cystine intake and inducing ferroptosis [61]. Nutlin-3, an inhibitor of the double-minute 2 homolog, increases p53 stability and maintains cellular GSH levels through a p53-21-dependent pathway, promoting cell survival under metabolic stress such as cystine loss [61]. On the other hand, p53 can inhibit the activity of dipeptidyl peptidase-4 (DPP4), blocking erastin-induced ferroptosis. Loss of p53 promotes the interaction between DPP4 and nicotinamide adenine dinucleotide phosphate oxidase 1 (NOX1), leading to the formation of the NOX1-DPP4 complex and mediating plasma membrane lipid peroxidation and ferroptosis [40]. NRF2 is a crucial regulatory factor that maintains intracellular redox homeostasis. It upregulates the expression of various genes involved in iron and ROS metabolism, such as NQO1, HO1, and FTH1, through the p62-Keap1-NRF2 pathway, inhibiting cellular ferroptosis [62]. Additionally, SLC7A11 has been identified as a transcriptional target of NRF2. Thus, other genes like SLC7A11 likely participate in the NRF2-mediated protection against ferroptosis, warranting further research [62]. Furthermore, a recent study discovered a novel function of vitamin K. The fully reduced form of vitamin K (Vitamin K hydroquinone, VKH2) acts as an antioxidant and effectively inhibits cell ferroptosis. The study also revealed that FSP1 is an efficient reductase that reduces vitamin K to VKH2, driving a novel non-classical vitamin K cycle. Therefore, vitamin K can effectively rescue cells and tissues from ferroptosis and serves as an efficient inhibitor of ferroptosis [63]. The pathways affecting the metabolic basis of ferroptosis are shown in Fig. 6.

#### Molecular mechanism of epigenetic modification involved in the regulation of ferroptosis

Epigenetic modifications refer to reversible and heritable changes in gene transcription achieved by adjusting chromatin states without altering DNA sequence. These modifications play a crucial role in various physiological and pathological processes [64]. Studies have found that epigenetic modifications such as histone modifications, DNA methylation, non-coding RNAs, etc., can activate or inhibit the transcription of genes like SLC7A11 and GPX4, regulate GSH synthesis, and lipids ROS production, thereby playing a vital role in the prevention and treatment of various diseases [65, 66]. In terms of histone methylation, histone methylation transferase-mediated histone methylation has been identified. Research has shown that histone methyltransferase G9a can catalyze the methylation of lysine 9 on histone H3, upregulating the expression of ferroptosis-related genes GPX4, SLC7A11, and SLC3A2, thereby inhibiting ferroptosis [65]. Histone H3K9 demethylase KDM3B, as a potential epigenetic regulator of ferroptosis, can upregulate the expression of the SLC7A11 gene and inhibit ferroptosis [67]. In terms of histone ubiquitination, Liu et al. reported that the deubiquitinase ovarian tumor protein 1 can directly interact and stabilize SLC7A11, thereby inhibiting ferroptosis [68]. Tumor suppressor p53 can recruit ubiquitin-specific protease 7 to reduce the proportion of H2Bub1 in the regulatory region of the SLC7A11 gene, leading to impaired GSH synthesis and accumulation of peroxidized lipids, making cells more sensitive to ferroptosis [69–71]. Additionally, BRCA1-associated protein 1 (BAP1), a deubiquitinase for H2A, significantly inhibits tumor occurrence and development. Zhang et al. demonstrated that BAP1 can reduce the level of H2Aub1 in the SLC7A11 promoter region, increase lipid peroxidation, and induce ferroptosis [72]. Regarding histone acetylation, NADPH oxidase plays a critical role in producing lipid ROS, and inhibitors of histone deacetylases can inhibit the accumulation of lipid ROS by suppressing the expression of NADPH oxidase genes [60, 73]. Notably, the acetylation-defective P53 mutant (P53 3KR) can indirectly inhibit the uptake of cysteine, decrease GSH production, and induce lipid ROS accumulation, leading to ferroptosis [67, 74]. Conversely, acetylation of histone H3 at lysine 27 in cancer tissue results in high expression of GPX4, rendering cells resistant to ferroptosis [67]. In terms of DNA/RNA methylation, Shen et al. discovered that m6A modification is upregulated, autophagy is activated, and ferroptosis is induced in induced pluripotent stem cells. Knockout of RNA methyltransferase 4 or overexpression of RNA demethylation-related genes can weaken the upregulation of m6A in induced pluripotent stem cells and inhibit ferroptosis [75]. In the aspect of non-coding RNAs, Zhang et al. found that miRNA-522 secreted by cancer-associated fibroblasts can target ALOX15 to block the accumulation of lipid ROS in gastric cancer cells and inhibit ferroptosis [76]. Studies have already shown that lncRNAs can regulate cellular oxidative stress and induce ferroptosis [77]. Wang et al. discovered that overexpression of the long non-coding RNA LINC00618 increases lipid ROS and blood lipid levels and enhances the expression of the ferroptosis-inducing factor ACSL4 [78]. LINC00336 is upregulated as a competitive endogenous RNA in lung cancer, and its deletion significantly increases intracellular iron, Fe2+, and lipid ROS levels, leading to ferroptosis [79]. Therefore, in-depth research on the regulatory mechanism of ferroptosis can help us better understand the mechanism of disease and seek new therapeutic targets.

## The role of ferroptosis key proteins on immune cells

### Effects of ferroptosis key proteins on CD4+ and CD8+ T cell subsets

**Fig. 7:**
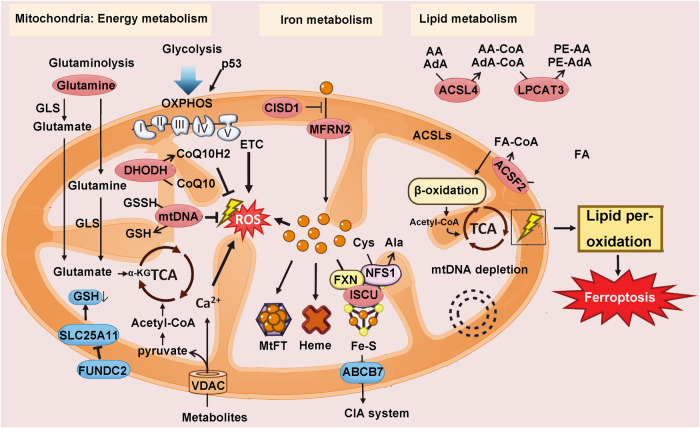
The mechanism of mitochondria in ferroptosis. This shows the association between mitochondria and ferroptosis. AA arachidonic acid, GSH glutathione, AdA adrenic acid.

The ferroptosis target Gpx4 plays a central role in maintaining cellular antioxidative homeostasis. Understanding the role of Gpx4 in immune cells can provide a better understanding of potential factors underlying immune cell ferroptosis [80]. Blood cells primarily originate from the bone marrow, where multipotent hematopoietic stem cells (including myeloid and lymphoid stem cells) differentiate into various hematopoietic progenitor cells, which subsequently develop into different lineages of mature blood cells under different conditions and locations [81]. Lymphoid stem cells enter the thymus through the bloodstream, where they undergo maturation into T cells. Subsequently, T cells are released and undergo proliferation and differentiation upon antigen stimulation [81]. HSCs possess several metabolic regulatory mechanisms to protect their functional integrity and counteract damage caused by genotoxic substances and stress-induced byproducts, thereby preventing bone marrow failure. The consequences of bone marrow failure are severe, leading to progressively reduced numbers of HSCs, decreased generation of mature blood cells, and diminished immune cell production. A recent study indicated that the absence of MYMS1 impairs the protective mechanism of hematopoietic stem cells against ferroptosis. In contrast to hematopoietic progenitor cells, hematopoietic stem cells are more susceptible to ferroptosis, possibly due to their lower protein synthesis rate [82]. Matsushita et al. investigated the impact of Gpx4 on peripheral T cell proliferation by establishing a lymphocyte-reduction-driven proliferation mouse model. Upon transferring wild-type and Gpx4-deficient T cells into the mouse model, while wild-type T cells proliferated normally post transplantation, Gpx4-deficient CD4+ and CD8+ T cells rapidly decayed within 7 days, undergoing ferroptosis, underscoring the innate role of Gpx4 in the proliferation and survival of CD4+ and CD8+ T cells [83]. The maintenance of peripheral T cell homeostasis depends on thymic output of T cells and self-proliferation of peripheral T cells [84]. T cell survival in a quiescent state requires a sub-threshold signal provided by self-MHC/peptide recognition and some cell-activating factors. Under certain conditions associated with lymphopenia, this self-MHC/peptide recognition provides a proliferative signal that leads to the proliferation of T cells and the generation of effector cells [85]. Additionally, ROS serve as important second messengers in T cell receptor (TCR) signaling for proliferation and effector function [86]. Lipid ROS and membrane ROS levels in CD4+ T cells cultured under different intensities of anti-CD3/CD28 stimulation are closely related. The sensitivity to lipid peroxidation and ferroptosis induced by different types of TCR and co-stimulatory signals also varies [87]. Therefore, Gpx4 is critical for T cell TCR responses and protects T cells from ferroptosis. Furthermore, CD8+ and CD4+ T cells lacking Gpx4 fail to proliferate and undergo ferroptosis after viral or parasitic infection. Interestingly, the lack of Gpx4 does not affect the peripheral homeostasis of memory CD4+ and CD8+ T cells or secondary T cell responses. This may be related to differences in metabolic activity between effector and memory cells, as effector cells utilize aerobic glycolysis for energy production, while memory cells rely on oxidative phosphorylation. Compared to effector cells, memory cells have greater mitochondrial spare respiratory capacity and produce fewer superoxides [88]. In addition, Wang et al. found that mammalian target of rapamycin complex 2 (mTORC2) is crucial for the long-term maintenance of virus-specific memory CD4+ T cells [89]. The mTORC2-AKT-GSK3β pathway may maintain the opening state of voltage-dependent anion channels on the mitochondrial membrane and maintain normal ROS levels in the mitochondria. Additionally, this pathway upregulates the expression of NRF2, which can upregulate GPX4 expression [90]. Gpx4 deficiency does not affect the proliferation of regulatory T cells (Tregs), which may be related to increased production of thioredoxin-1 in Tregs, enhancing their tolerance to oxidative stress [83, 91]. This indicates that different T cell subsets have different requirements for Gpx4. Tregs are a subset of T cells that control the body’s autoimmune responses. Tregs, typically referred to as CD4 + CD25+Foxp3+ T cells, can survive in tumor microenvironments with persistent oxidative stress. The increased proportion of Tregs in tumors is often associated with poor prognosis and low survival rates in various cancer patients. Specific deletion of Gpx4 in Tregs impairs their survival in tumors, increases infiltration of T cells into tumors, and enhances antitumor immune responses, indicating that Gpx4 maintains the survival and immunosuppressive functions of Tregs to promote tumor immune evasion [92]. Follicular helper T cells (Tfh) are involved in germinal center formation and B cell differentiation. Tfh cells often receive signals from TCR, CD28, and PD-1 when interacting with B cells in germinal centers, leading to increased cellular and lipid ROS and increased sensitivity to ferroptosis. Deletion of Gpx4 results in ferroptosis and loss of germinal center responses in Tfh cells [87]. In terms of iron metabolism and homeostasis in immune cells, a study revealed that iron overload promotes abnormal differentiation of pathogenic T cells, including follicular helper T cells, by regulating DNA demethylation, thus exacerbating autoantibody production and the development of systemic lupus erythematosus (SLE) [26]. This suggests that intracellular iron ions in T cells may serve as a new therapeutic target for the treatment of SLE. The mechanism of mitochondria in ferroptosis is summarized in Fig. 7.

In summary, T cell responses, including proliferation and immune reactions, depend on the TCR. ROS play a critical role in T cell physiology and are closely associated with TCR signaling. Gpx4, an antioxidant enzyme, protects T cells from excessive ROS-induced oxidative stress and prevents iron-mediated cell death. Gpx4 is required for the proliferation of T cells, their primary immune responses, and the participation of Tfh cells in germinal center reactions, as these processes are susceptible to iron-induced death. However, the dependence on Gpx4 is not essential for Treg proliferation, peripheral homeostasis of memory T cells, and secondary T cell responses. Different T cell subsets may have distinct requirements for Gpx4, which could be attributed to their diverse metabolic activities or unique antioxidant mechanisms.

### The role of ferroptosis key proteins on B cells

B cells undergo maturation in the bone marrow, during which they enter an antigen-independent phase. Subsequently, they migrate from the bone marrow to the blood, and further transition into transitional 1B and transitional 2B cells before reaching the spleen or lymph nodes. In mice, transitional 2B cells differentiate into follicular B cells and marginal zone B cells in the spleen, while B1 cell differentiation occurs in the bone marrow. Antibody production by follicular B cells is T cell-dependent, whereas B1 and marginal zone B cells rapidly respond to blood-borne antigens in a T cell-independent manner [93]. Muri et al. investigated the survival of different B cell subtypes in the absence of Gpx4. They found that follicular B cells, aided by Tfh cells, undergo germinal center reactions and generate high-affinity antibody responses. The development, homeostatic proliferation, germinal center reactions, and antibody responses of follicular B cells were not affected by Gpx4 deficiency [94]. However, Gpx4 is indispensable for the development, maintenance of homeostasis, and antibody responses against Streptococcus pneumoniae in B1 and marginal zone B cells. This may be attributed to the distinct metabolic processes of B1 and marginal zone B cells compared to follicular B cells. B1 and marginal zone B cells heavily rely on fatty acid uptake to sustain their metabolic functions, and the accumulation of excessive fatty acids under Gpx4 deficiency leads to heightened oxidative stress and increased susceptibility to iron-mediated cell death [94].

### The role of ferroptosis key proteins on macrophages

Tissue-resident macrophages can differentiate from monocytes released from the bone marrow or be generated during embryonic development. Piattini et al. performed Gpx4 knockout in mouse myeloid immune cell subpopulations, and the development and homeostasis of tissue-resident macrophages in the lung, peritoneum, spleen, and bone marrow (considered as M0 state) were unaffected [95]. Induction of Gpx4-deficient macrophages with IL-4, which triggers M2 macrophage activation, severely disrupted the cellular redox balance and led to ferroptosis. However, this response was not observed in M1 macrophages, which were able to sustain cell survival and their antimicrobial functions. Stimulation with lipopolysaccharide (LPS) and IFNγ not only activated M0 macrophages into M1 phenotype but also upregulated the expression of inducible nitric oxide synthase (iNOS) in Gpx4-deficient macrophages. This upregulation of iNOS induced an increase in nitric oxide (NO) expression, equipping the cells with resistance against ferroptosis. Overall, Gpx4 plays a critical protective role in the proliferation and function of immune cells, although certain immune cell subsets may not rely heavily on Gpx4 protection, possibly due to their distinct energy metabolic activities and antioxidant mechanisms. Furthermore, Gpx4 knockout in regulatory T cells can suppress tumors, while selenium supplementation can enhance the survival of Tfh cells and improve immune capacity [95]. Therefore, Gpx4 may serve as a therapeutic target in immune cells for the treatment of various diseases.

## Ferroptosis affects immune cell function

### Ferroptosis releases injury-associated molecular patterns and affects immune cell function

Ferroptosis has been postulated as a type of immunogenic cell death triggered by stress factors, prompting adaptive immune reactions against antigens from deceased cells. Apart from stimulating immune responses via death-associated antigens, immunogenic cell death is accompanied by the release of diverse damage-associated molecular patterns (DAMPs), such as calreticulin (CRT), high mobility group protein 1 (HMGB1), adenosine triphosphate (ATP), heat shock proteins (HSPs), and others. These DAMPs can engage with pattern recognition receptors on antigen-presenting cells, instigating a sequence of cytokine generation and immune activation [96]. Efimova et al. delved into the immunogenic nature of ferroptosis in their research, discovering that early-stage ferroptotic cells with intact membranes display heightened immunogenicity compared to cells in later stages of ferroptosis. ATP and HMGB1 stand out as distinctive DAMPs involved in immunogenic cell demise. Early-stage ferroptotic cells express calreticulin, which, upon binding to the LRP1 receptor on dendritic cells, fosters the phenotypic maturation of bone marrow-derived dendritic cells, eliciting vaccine-like effects in immunocompetent mice. This suggests a potential role for ferroptosis in tumor immunotherapy. Suppressing extracellular ATP functions by impeding the P2X7 receptor with 2’,3’-dialdehyde ATP diminishes the recruitment of antigen-presenting cells and anti-tumor immune responses. These DAMPs provoke immune reactions against cancerous cells, primarily released during the initial phases of ferroptosis [97, 98]. Liu et al. treated THP-induced macrophages co-cultured with human bronchial epithelial cells with cigarette smoke extract, observing elevated levels of nuclear receptor coactivator 4 (NCOA4). NCOA4, involved in ferritinophagy, plays a critical role in the selective autophagy of ferritin, contributing to ferroptosis by enhancing ferritin degradation and triggering the Fenton reaction in human bronchial epithelial cells [99]. Upregulation of NCOA4 also increases the ratio of M2/M1 macrophages and levels of matrix metalloproteinases, which are factors involved in inducing emphysema. However, direct evidence of how NCOA4 upregulation induces macrophage polarization in relation to ferroptosis remains to be demonstrated. Nevertheless, significant increases in DAMPs (including IL-33 and HMGB1) were observed in bronchoalveolar lavage fluids, with IL-33 promoting M2 polarization and HMGB1 inducing M1 polarization through the advanced glycation end-products receptor pathway. Other DAMPs may also be involved in macrophage polarization. Wen et al. studied the release mechanism of HMGB1 from ferroptosis cells and found that HMGB1 can be released by ferroptosis cells through an autophagy-dependent pathway. Specifically, autophagy-mediated histone deacetylase inhibition promotes acetylation of HMGB1, leading to its release during ferroptosis [100]. HMGB1 triggers an inflammatory response in macrophages through the AGER pathway, resulting in the release of TNF. Antibodies against HMGB1 or AGER can weaken the inflammatory response induced by ferroptosis cells. Additionally, one study indicated that HMGB1 can upregulate heme oxygenase 2 and transferrin expression through the RAS-JNK/p38 pathway, promoting erastin-induced ferroptosis [101]. Liu et al. developed Gpx4 knockout mice and initiated acute pancreatitis with cerulein, investigating ferroptosis-related DAMPs using an antibody chip. They observed early release of decorin (DCN) in early-stage ferroptosis, associated with an autophagy-driven protein secretion process [102]. DCN is released from early-stage ferroptotic cells via lysosomal exocytosis and damaged plasma membranes, binding to AGER on macrophages. This interaction prompts M1 polarization and triggers pro-inflammatory cytokine production, exacerbating acute pancreatitis through the NFKB/NF-κB pathway. Antibodies against DCN or AGER shielded Gpx4 knockout mice from cerulein-triggered pancreatitis, suggesting an indirect influence of ferroptosis-released DAMPs on immune cells [102]. The cellular and molecular mechanisms underlying ischemia-reperfusion-induced myocardial inflammation after heart transplantation are not well understood. Li et al. found that neutrophils exhibit reduced velocity and increased adhesion to vascular walls during ischemia-reperfusion. Mechanistically, ferroptosis in transplanted hearts triggers the initial release of DAMPs, which bind to endothelial Toll-like receptor 4 (TLR4) and stimulate type I interferon production via Trif-mediated signaling, promoting the recruitment of neutrophils to the injured heart [103]. Further research is needed to identify the main participants and executors of inflammation induced by ferroptosis during ischemia-reperfusion after heart transplantation. In summary, ferroptosis releases DAMPs distinct from apoptosis, with early-stage ferroptosis exhibiting stronger immunogenicity. Cell death-associated DAMPs released during ferroptosis include CRT, ATP, HMGB1, and IL-33, which can promote the maturation and recruitment of antigen-presenting cells, as well as induce macrophage polarization. Additionally, DAMPs can stimulate endothelial cells to produce interferons and recruit neutrophils.

### Ferroptosis releases other molecules that affect immune cell function

Ferroptosis is characterized by the accumulation of peroxidized lipids, particularly phosphatidylethanolamine with arachidonic or adrenic acid acyl groups [104]. The peroxidized lipids in ferroptosis may interact with the immune system as Luo et al. [105]. found that ferroptosis cells can be engulfed by phagocytic cells. They discovered that phosphatidylethanolamine peroxidation, especially SAPE-OOH (1-steaoryl-2-15-HpETE-sn-glycero-3-phosphatidylethanolamine), which is abundantly present on the membrane of ferroptosis cells, serves as a critical signal for engulfment. The Toll-like receptor 2 (TLR2) on macrophages recognizes SAPE-OOH and promotes macrophage phagocytosis. They observed that during the early stages of engulfment of ferroptosis cells, typical “eat me” signals such as phosphatidylserine and peroxidized phosphatidylserine were absent from the cell membrane [106]. Ma et al. treated macrophages infected with Staphylococcus aureus, Escherichia coli, and Salmonella enterica serovar Typhimurium with ferroptosis inducers, finding that these agents assisted macrophages in combating intracellular bacteria [107]. Mechanistically, during the early stages of bacterial infection, cells elevate unstable intracellular iron levels by activating two iron metabolism pathways: nuclear factor erythroid 2–related factor 2/heme oxygenase-1 (Nrf2/HO-1) and ferritin/NCOA4. Nrf2, a nuclear transcription factor, translocates to the cell nucleus to upregulate HO-1, converting heme to biliverdin, iron, and CO [108]. Dai et al. discovered that oxidative stress-induced autophagy-dependent ferroptosis in cancer cells leads to the packaging and release of KRASG12D into exosomes, subsequently engulfed by macrophages through an AGER-dependent mechanism [109]. AGER-mediated STAT3 activation induces fatty acid oxidation, causing long-chain fatty acids to break down into acetyl-CoA, polarizing macrophages into an M2-like tumor-promoting phenotype. Inhibiting the release and uptake of KRASG12D can suppress macrophage-mediated tumor growth in pancreatic cancer [109]. Additionally, the expression levels of KrasG12D in macrophages are correlated with poor survival in pancreatic cancer patients, offering new targeted anticancer strategies against KRAS. Research reveals that iron overload promotes Tfh cell expansion, pro-inflammatory cytokine secretion, and autoantibody production in lupus-prone mice. Mice treated with HID exhibit increased percentages of Tfh cells and antigen-specific GC responses [26]. Iron supplementation promoted Tfh cell differentiation, while iron chelation inhibited Tfh cell differentiation. miR-21/BDH2 pathway was found to promote iron accumulation during Tfh cell differentiation, further enhancing Fe2+ -dependent TET enzyme activity and BCL6 gene demethylation [26]. Thus, maintaining iron homeostasis may be critical for eliminating pathogenic Th cells and may contribute to improving the management of SLE patients. In conclusion, it is evident that cell death in ferroptosis can release various molecules that influence immune cell differentiation, phagocytosis, microbial killing, and other immune responses, thereby affecting the progression of related diseases. Therefore, these molecules and their activated pathways may serve as therapeutic targets for specific diseases.

## Immune cells affect ferroptosis

### CD8+ T cells affect ferroptosis

Wang et al. discovered that activated CD8+ T cells in the process of tumor immunotherapy can enhance the specific lipid peroxidation level of ferroptosis in tumor cells [110]. After blocking the ferroptosis pathway within tumor cells, their sensitivity to immunotherapy is lost. Further investigation revealed that the release of IFN-γ by CD8+ T cells downregulates the expression of SLC3A2 and SLC7A11, both of which are subunits of the glutamate-cystine antiporter system Xc-. This impairs the uptake of cystine by tumor cells, leading to increased lipid peroxidation and ferroptosis. Liao et al. further found that IFN-γ released by CD8+ T cells synergizes with arachidonic acid to effectively induce cellular ferroptosis in various tumor cell lines [110]. During tumor immunotherapy, IFN-γ released by activated CD8 + T cells also upregulates the expression of ACSL4 in tumor cells through the STAT1-IRF1 signaling pathway [111]. Targeted phospholipid analysis revealed that arachidonic acid preferentially incorporates into phospholipids containing C16 and C18 acyl chains, which are common fatty acids found in the blood, with oleic acid enhancing the lipid species of arachidonic acid-d5-bound PE and PC in tumor cells. LPCAT3 and LOX are involved in the integration of arachidonic acid into the membrane phospholipids and the oxidation of these phospholipids, respectively, promoting IFN-γ and arachidonic acid-induced ACSL4-dependent tumor ferroptosis [112]. The regulation of ferroptosis by CD8 + T cells in tumor immunotherapy reveals that the ferroptosis pathway can be modulated by T cells, and the immune system can suppress tumor development through cancer cell ferroptosis. IFN-γ is mainly produced by T cells, NK cells, and NK T cells, and it is a pleiotropic cytokine with diverse functions. While IFN-γ can induce tumor cell killing, it can also promote tumor dormancy, edit tumor cells to cause immune evasion, and contribute to tumor relapse [113]. Recent studies have shown that IFN-γ can downregulate SLC3A2 and SLC7A11 while upregulating ACSL4, promoting tumor cell ferroptosis. Therefore, increasing the sensitivity of tumor cells to ferroptosis induction may enhance the effectiveness of tumor immunotherapy.

### Macrophages affect ferroptosis

Macrophages can release various molecules that affect ferroptosis. Kapralov et al. found that M1 macrophages exhibit stronger resistance to ferroptosis compared to M0 and M2 macrophages. They discovered that M1 macrophages have higher levels of inducible nitric oxide synthase (iNOS or NOS2), leading to increased production of NO [114]. NO can inhibit 15-lipoxygenase, similar to the strength of GPX4 in resisting ferroptosis. Moreover, NO possesses membrane diffusibility, granting surrounding cells near M1 macrophages the ability to resist ferroptosis. Pseudomonas aeruginosa utilizes 15-lipoxygenase released in vesicles to oxidize host arachidonic acid phosphatidylethanolamine into ferroptotic death-inducing 15-hydroperoxy-arachidonic acid phosphatidylethanolamine, triggering ferroptosis in epithelial cells. Concurrently, it degrades host Gpx4 through lysosome-mediated autophagy. Co-culture models confirmed that M1 macrophages release NO to remotely protect epithelial cells from Pseudomonas aeruginosa-induced ferroptosis [115, 116]. The inhibitory effect of NO on ferroptosis in epithelial cells is achieved by diffusing to the catalytic site of 15-lipoxygenase isoform 2 to suppress phospholipid peroxidation. Additionally, adipose tissue macrophages secrete miR-140-5p in extracellular vesicles, which targets SLC7A11 in cardiomyocytes, inhibiting ferroptosis induced by reduced glutathione synthesis, presenting a new therapeutic strategy for obesity-induced cardiac injury [117]. Itaconate is a metabolite synthesized by cis-aconitate decarboxylase and is produced by lipopolysaccharide-activated macrophages through the diversion of cis-aconitate from the tricarboxylic acid cycle. In macrophages, 4-octyl itaconate, a cell-permeable derivative of endogenous itaconate, inhibits Nrf2 degradation pathway and promotes transcription of target genes, including SLC7A11, glutathione-cysteine ligase, and Gpx4, alleviating sepsis-induced acute lung injury [118]. Additionally, itaconate can activate NOCAD 4-mediated ferritin deposition, thereby inducing ferroptosis in Nrf2-deficient cell lines [119]. Wu et al. observed increased extracellular trap formation by macrophages (macrophage extracellular traps, METs) and ferroptosis in patients undergoing hepatic resection with hepatic inflow occlusion as well as in mice subjected to hepatic ischemia-reperfusion injury [120]. To elucidate the role of METs in ferroptosis of hepatocytes during ischemia-reperfusion injury, they utilized a co-culture model of macrophages exposed to hypoxia/reoxygenation along with hepatocytes. Following hypoxia/reoxygenation, macrophage METs release increased, leading to ferroptosis in hepatocytes, which could be reversed by Cl-amidine, an METs inhibitor. Tumor-associated macrophages (TAMs) are a major component of the tumor microenvironment directly influencing tumor cell growth, angiogenesis, and immune suppression. Research has found that TAM-derived TGF-β1 regulates the expression of hepatic leukemia factor (HLF) by modulating SMAD3, resulting in transactivation of gamma-glutamyltransferase 1 (GGT1). GGT1 catalyzes the breakdown of extracellular glutathione into intracellular cysteine, enhancing Gpx4 activity and promoting resistance to tumor cell ferroptosis. Conversely, breast cancer cells produce IL-6, which activates the JAK2/STAT3 axis, inducing TAMs to secrete TGF-β1, thereby forming a positive feedback loop that ultimately facilitates malignant tumor progression [121]. However, other studies have shown that TGF-β1, through Smad3 activation, inhibits the expression of the glutamate-cystine antiporter system Xc-, enhancing lipid peroxidation levels in PLC/PRF/5, Huh7, Huh6, and Hep G2 cells marked by early TGF-β1 genes, but not in cells marked by late TGF-β1 genes [122]. In summary, different types of macrophages can produce various molecules that affect ferroptosis, including NO, extracellular vesicles, itaconate, TGF-β1, etc.

### Neutrophils affect ferroptosis

The mechanism underlying the exacerbation of intracerebral hemorrhage (ICH) in diabetes is unclear. In a mouse model of streptozotocin-induced diabetes with hyperglycemia, high glucose not only increased neutrophil infiltration but also impaired the activity of peroxisome proliferator-activated receptor γ (PPARγ), a transcription factor for lactoferrin (Ltf) encoded by the PPARγ gene. Ltf, when taken up by cells through its receptor, reduces intracellular Fe concentration. Decreased secretion of Ltf leads to increased intracellular Fe concentration in neuronal cells, thereby exacerbating neuronal cell ferroptosis [123]. Supplementing Ltf or inhibiting neuronal ferroptosis provides a potential avenue for improving the prognosis of acute diabetes-related ICH. However, the mechanisms by which high glucose impairs PPARγ activity and leads to neutrophil activation require further elucidation. The degree of tumor necrosis is negatively correlated with the survival rate of patients with glioblastoma multiforme (GBM). However, the nature and mechanisms driving tumor necrosis remain unclear. Yee et al. scrutinized the involvement of neutrophils in tumor necrosis within a mouse model of glioblastoma (GBM) steered by the PDZ-binding motif [124]. GBM showcases tumor cell necrosis, wherein substances released during this process, like damage-associated molecular patterns (DAMPs), spur neutrophil infiltration, particularly at recently necrotic margins [124]. These mobilized neutrophils convey myeloperoxidase (MPO) to tumor cells either directly or through close cell-to-cell interaction, instigating lipid peroxidation within tumor cells and fostering tumor cell ferroptosis [124]. Specific tumor traumas encountered during initial tumor advancement attract neutrophils to the injury site, subsequently prompting tumor cell ferroptosis, initiating a favorable feedback mechanism that amplifies the evolution of GBM necrosis [124].

## Iron metabolism, ferroptosis in autoimmune diseases

### SLE

SLE is an autoimmune disease characterized by the production of self-antibodies, sustained inflammation, and multi-organ damage. Epidemiological studies have shown that the prevalence of SLE is close to or even exceeds 50–100 per 100,000 people. The onset and prognosis of lupus are influenced by multiple factors, including genetics, natural environment, and social factors [125]. SLE, as a complex autoimmune disease, is characterized by aberrant expansion of pathogenic T cells, which plays a crucial role in disease development. SLE patients often exhibit impairments in iron transport and utilization. Research has shown that SLE patients have increased hypochromic areas in red blood cells and significantly reduced iron levels within and outside the bone marrow, while bone marrow proliferation remains normal [126]. Recent research has discovered that disruptions in iron metabolism and ferroptosis mediate the pathogenesis of SLE. Blocking iron uptake receptors in a mouse model of SLE was found to reduce disease pathology and promote the activity of anti-inflammatory regulatory T cells. Targeting iron metabolism in immune system cells may provide a novel approach for treating SLE [126].

In terms of iron metabolism, researchers investigating T cell metabolism in lupus noticed that iron appeared to be the “culprit” behind multiple T cell problems. Interestingly, despite lupus patients commonly having anemia, their T cells exhibit high iron levels. To explore T cell iron metabolism in lupus, researchers used CRISPR gene editing to assess iron-handling genes in T cells. They identified the transferrin receptor (TFR/CD71) responsible for iron uptake as crucial for promoting inflammatory T cell activity while inhibiting anti-inflammatory regulatory T cell activity. The researchers found that the expression of transferrin receptors was higher in T cells from both susceptible lupus-prone mice and lupus patients, resulting in the accumulation of excessive iron and impaired mitochondrial function, as well as alterations in other signaling pathways. Blocking the transferrin receptor with specific antibodies reduced intracellular iron levels, suppressed inflammatory T cell activity, and enhanced regulatory T cell activity. Treating lupus-prone mice with these antibodies reduced kidney and liver damage and increased the production of the anti-inflammatory factor IL-10. In the T cells of lupus patients, the expression of transferrin receptors was correlated with disease severity, and blocking this receptor in vitro enhanced IL-10 production. Based on the above research findings, future studies will aim to develop T cell-specific antibodies targeting the transferrin receptor to avoid potential off-target effects (as the transferrin receptor mediates iron uptake in many cell types). Furthermore, the discovery that blocking the transferrin receptor enhances regulatory T cell activity warrants further exploration. Iron homeostasis has recently been identified as a potential target for improving SLE. A study revealed that iron overload promotes aberrant differentiation of pathogenic T cells, primarily Tfh cells, through DNA demethylation, exacerbating autoantibody production and SLE pathogenesis. Intracellular iron levels in CD4 + T cells of SLE patients were found to be significantly elevated and positively correlated with the percentage of Tfh cells. Experimental evidence using a high-iron diet demonstrated that increased iron levels favored Tfh and GCB cell expansion, enhanced secretion of inflammatory cytokines IFN-γ and IL-17A by CD4 + T cells, promoted autoantibody production in MRL/lpr lupus mice, and worsened their disease phenotype. Iron supplementation showed induction of Tfh cell differentiation in vitro. Conversely, reducing intracellular iron accumulation using 2,5-Dihydroxybenzoic acid and iron chelator CPX significantly inhibited Tfh cell differentiation. Mechanistically, the study revealed the involvement of the miR-21/BDH2 pathway in regulating intracellular iron accumulation and Tfh cell differentiation. Upregulation of miR-21 or interference with BDH2 expression enhanced TET protein activity, resulting in decreased DNA methylation of the BCL6 gene promoter, ultimately promoting BCL6 gene expression [26]. In terms of ferroptosis, a research demonstrated that neutrophil ferroptosis in SLE patients was induced by the synergistic effect of autoantibodies and IFN-α, leading to transcriptional repression factor CREMα binding to GPX4 promoter and subsequent downregulation of GPX4 protein expression. In vivo studies showed that specific deletion of myeloid cell Gpx4 in mice exhibited SLE-like clinical manifestations, which could be alleviated by treatment with liproxstatin-1, an ferroptosis inhibitor, and attenuated disease progression. Both SLE patient serum and RSL-3 treatment significantly reduced the viability of normal neutrophils, while treatment with liproxstatin-1 (LPX-1) and iron chelator deferoxamine (DFO) rescued neutrophil death induced by SLE patient serum, suggesting that ferroptosis is the major form of neutrophil death in SLE [27]. The authors also conducted experiments to exclude the possibility of LPX-1 inhibiting NETosis and affecting IgG or IFN-α production [27]. The above findings demonstrate that ferroptosis is a crucial driving factor in neutrophil death in SLE. Concentration-dependent increases in lipid-associated ROS of neutrophils were observed with the addition of SLE IgG or IFN-α in normal serum, which could be reversed by depleting IgG or antagonizing IFN-α receptor. Both IFN-α and SLE IgG induced neutrophil ferroptosis. The above results indicate that ferroptosis induced by the combined action of IFN-α and IgG is the major form of neutrophil death in the serum environment of SLE patients. Subsequently, the authors analyzed the occurrence of neutrophil ferroptosis in different lupus-prone mouse models. Consistent with SLE patients, MRL/lpr and NZB/W F1 mice exhibited reduced neutrophil viability and increased lipid-associated ROS levels. Furthermore, suppressing lipid-associated ROS production in neutrophils by LPX-1 treatment in MRL/lpr mice effectively attenuated disease progression, decreased production of autoantibodies and various inflammatory cytokines, increased serum C3 complement levels, and reduced the severity of splenomegaly, lymphadenopathy, and lupus nephritis. These results indicate that neutrophil ferroptosis is the main cause of neutrophil depletion in lupus, and targeting neutrophil ferroptosis can be an effective therapeutic strategy for treating SLE.

To further investigate the relationship between neutrophil GPX4 expression and SLE pathogenesis, the researchers generated specific myeloid cell Gpx4 knockout mice (Gpx4^fl/wt^ LysMCre^+^). Compared to Gpx4^fl/fl^ mice, the neutrophils of Gpx4^fl/wt^ LysMCre^+^ mice exhibited significantly reduced viability, which could be restored by treatment with the ferroptosis inhibitor LPX-1. Gpx4^fl/wt^ LysMCre^+^ mice also displayed SLE-like clinical manifestations, including alopecia, lymphadenopathy, splenomegaly, proteinuria, significant increases in anti-dsDNA antibodies, IFN-α, IL-6, and decreased complement C3 levels, indicating a close association between decreased neutrophil GPX4 protein expression and SLE pathogenesis. Lastly, the study elucidated the mechanism by which IFN-α and SLE IgG induce neutrophil ferroptosis, namely, by promoting the binding of CREM to the Gpx4 promoter, leading to downregulation of GPX4 expression. Consequently, targeted inhibition of neutrophil ferroptosis holds promise as a novel therapeutic strategy for treating SLE [127].

**Fig. 8:**
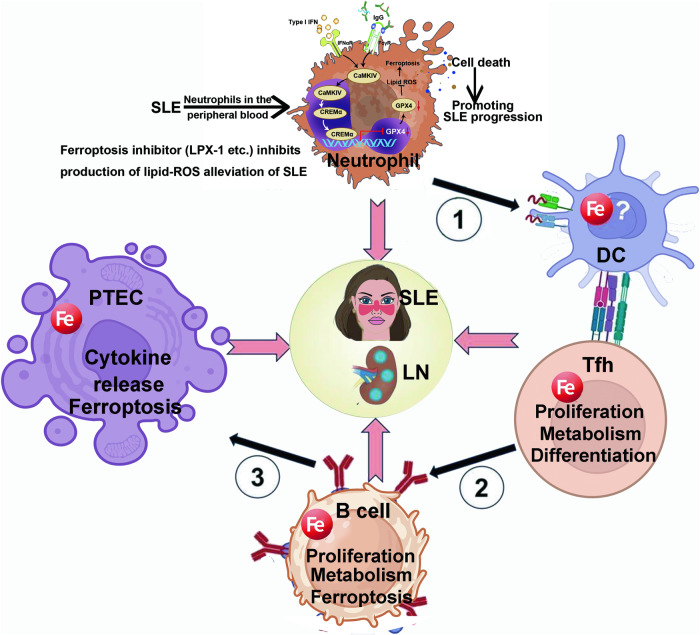
The mechanism of ferroptosis in SLE. Ferroptosis is involved in the development of SLE. DC dendritic cells, PTEC proximal tubular epithelial cells, Tfh follicular helper T cells.

In summary, this study demonstrates that neutrophil ferroptosis is a significant factor causing neutrophil depletion and triggering SLE. Targeted inhibition of neutrophil ferroptosis may represent a promising and effective therapeutic strategy for treating SLE (Fig. 8).

### Lupus nephritis (LN)

LN is one of the most common and severe organ manifestations in SLE and is a significant contributor to disability and mortality in SLE patients [128]. Within 15 years of diagnosis, 10–30% of LN patients progress to end-stage renal disease, which is a major cause of death in SLE [129]. Recent studies have indicated that susceptibility genes for LN (disrupting immune tolerance) can enhance the innate immune signaling pathway, promoting lymphocyte activation and leading to kidney damage [130]. The dysregulation of cell death and defective clearance of dying cells are closely associated with the pathogenesis of LN [131]. By evaluating different cell programmed death scores in proliferative nephritis, it has been found that ferroptosis in the glomerular compartment of LN patients is significantly and specifically increased compared to other forms of programmed cell death in renal diseases. Furthermore, the ferroptosis score is intricately associated with blood urea nitrogen, SLE disease activity index, serum creatinine, and complement 4, and negatively correlates with glomerular filtration rate [132]. Additionally, enhanced iron metabolism and reduced fatty acid synthesis may be the most important factors contributing to ferroptosis within the glomerulus. Analysis of single-cell sequencing datasets, along with immunohistochemistry and immunofluorescence staining validation, have revealed that abnormally activated lipid peroxidation in CD163+ macrophages and CD10 + PC+ (pyruvate carboxylase) epithelial cells suggests their potential involvement in ferroptosis within the glomerular compartment [132]. Moreover, excess production of ROS and abnormal infiltration of immune cells have been shown to be associated with LN induced by ferroptosis [133].

In terms of renal parenchymal cells in lupus nephritis, Alli et al. found increased lipid peroxidation and increased acyl-CoA synthetase long-chain family member 4 (a pro-ferroptosis enzyme) in the renal tubules of lupus nephritis patients and mice. Renal inflammation reduces the expression of SLC7A11, a cysteine transporter, and impairs the glutathione synthesis pathway, resulting in low expression of glutathione peroxidase 4 (a ferroptosis inhibitor). Lipidomics of nephritic kidneys confirmed the presence of ferroptosis. Using nephrotoxic serum, immune complex glomerulonephritis was induced in syngeneic mice, demonstrating that impaired iron sequestration in proximal tubules exacerbates ferroptosis. Serum from lupus nephritis patients made human proximal tubule cells more susceptible to ferroptosis, which was inhibited by a novel ferroptosis inhibitor, Liproxstatin-2. In summary, they have identified renal ferroptosis as a pathological feature and contributing factor of tubular injury in human and mice lupus nephritis [134]. In the context of pertinent biomarkers, the expression of 4-HNE exhibited a notable increase in both the glomeruli and tubulointerstitium. Transcriptomically, 19 FR-DEGs in the glomeruli and 15 FR-DEGs in the tubulointerstitium (comprising genes related to iron metabolism, inhibitors of the antioxidant system, and inhibitors of ferroptosis) displayed substantial alterations in LN. Within these, LTF, CYBB, and CCL5 manifested upregulation in both glomeruli and tubulointerstitium of LN, whereas G0S2 and AKR1C1 showed downregulation [135]. A recent investigation utilizing single-cell RNA sequencing and flow cytometry unveiled a subset of neutrophils with elevated IL-6 expression, correlated with IL-6 receptor and SLC7A11 expression in B cells of lupus nephritis. Additionally, neutrophils in lupus-afflicted kidneys supplied IL-6 via SLC7A11 to boost B cell resistance against ferroptosis; suppressing SLC7A11 markedly heightened B cell ferroptosis while reducing B cell proliferation. This exploration sheds light on the interaction between neutrophils and B cells in the progression of lupus nephritis [136]. In terms of transcription factor modulation, the central gene ATF3 potentially contributes to inflammation and immune injury in LN by engaging in ferroptosis mechanisms [137]. Wu et al. have identified PTEN and NR4A1 as ferroptosis-related genes that potentially serve as diagnostic biomarkers for lupus nephritis [138]. A recent study highlighted the occurrence of ferroptosis in CD4 + T cells of SLE patients, showing elevated expression of ferroptosis-related genes ACSL4 and SLC7A11 alongside reduced NRF2 expression. Interference with or inhibition of SLC7A11 dampened CD4 + T cell activation, whereas SLC7A11 overexpression enhanced the activation of CD4 + T cells. The reinstatement of CD4 + T cell activation impeded by iron chelation was achieved through supplementation with N-acetylcysteine. N-acetylcysteine supplementation stimulated CD4 + T cell activation and facilitated their differentiation into Th1 and Tfh subsets in mice [139]. Exploring iron metabolism further unveiled that the miR-21/BDH2 pathway enhances Fe2 +-dependent TET enzyme activity in Tfh cells, resulting in heightened hydroxymethylation levels in the BCL6 gene promoter region and reduced methylation of the BCL6 gene promoter region [140].

In a mouse model, a study found increased levels of non-heme iron in the kidneys of New Zealand Black/White (NZB/W) lupus nephritis mice compared to age-matched healthy New Zealand White (NZW) mice. Biodistribution studies showed that the presence of iron bound to transferrin in NZB/W mouse kidneys correlated with increased urinary protein, while non-transferrin-bound iron or ferritin had no effect on urinary protein levels. The excretion rate of transferrin in NZB/W mice significantly increased with the production of urinary protein, indicating increased tubular exposure and potential tubular reabsorption. Compared to NZW mice, the expression of the transferrin receptor 24p3R in the renal tubules of NZB/W mice was reduced, while transferrin expression and ferritin expression were increased, consistent with increased iron accumulation and compensatory downregulation of uptake pathways. Treatment of NZB/W mice with an iron chelator deferiprone significantly delayed the onset of albuminuria and reduced blood urea nitrogen levels. These findings suggest the pathological changes in iron homeostasis in lupus nephritis, contributing to the development of renal damage [141]. Hepcidin, a major iron regulatory and endogenous ferroptosis protective molecule, has been shown to decrease the availability of free iron, reduce macrophage and T cell infiltration in the kidneys, and further improve renal inflammation, thus alleviating the severity of lupus nephritis in susceptible mouse models. Therefore, inhibiting ferroptosis may be a therapeutic option for lupus nephritis [142].

In summary, the findings from the above studies suggest that iron-catalyzed reactive oxygen species may contribute to the accumulation of lipid hydroperoxides in proximal tubular epithelial cells. These iron-catalyzed oxidants can further enhance inflammation transcription factors induced by protein and autoantibodies, leading to the production of matrix proteins, cytokines/chemokines, and immune cell infiltration. The increased glomerular permeability and subsequent interactions between tubular injury, tubulointerstitial inflammation, and the progression of lupus nephritis (LN) towards renal dysfunction result in additional tissue damage in lupus. In future research, it is anticipated that investigations targeting ferroptosis in lupus nephritis will focus on iron metabolism, amino acid metabolism, lipid metabolism, and other metabolic pathways. These studies hold promise for the development of novel therapeutic strategies targeting iron metabolism and ferroptosis, thus paving the way for new avenues of research in lupus nephritis.

### Rheumatoid arthritis (RA)

RA is an autoimmune disease characterized primarily by erosive arthritis. Its pathological basis is synovitis, initially manifested as morning stiffness, swelling, and pain in small joints such as hands and feet, which can progress to joint deformities and disability [143]. The disease commonly occurs in middle-aged individuals, with a global incidence rate of approximately 1% [144]. Within the first 2-3 years of onset, the disability rate in untreated patients can reach 70%. Currently, RA cannot be cured and is often referred to as the “undying cancer,” significantly impacting the quality of life for affected individuals [145]. Xia et al. found a significant correlation between the occurrence of rheumatoid arthritis and the aberrant expression of disease-associated genes related to ferroptosis. These characteristic genes induce the development of the disease by influencing relevant signaling pathways, as identified through analysis of the competing endogenous RNA (ceRNA) network mediated by long non-coding RNAs associated with rheumatoid arthritis. This analysis has allowed for the identification of potential therapeutic targets and signaling pathways [146, 147]. Studies have shown an increase in lipid peroxidation levels in the serum and synovial fluid of RA patients, along with alterations in the antioxidant system [148]. Therefore, excessive production of reactive oxygen species (ROS) is more likely to inhibit osteoblast differentiation and lead to bone destruction. Low concentrations of iron ions promote the growth of osteoblast precursor cells (MC3T3-E1), while high concentrations of iron ions inhibit their growth and increase ROS levels. Excessive iron ions can activate the p38-MAPK pathway and block the PI3K/AKT and JAK/STAT3 signaling pathways, thereby inducing death in MC3T3-E1 cells [149]. Iron overload can partially inhibit the activity of osteoblasts, thereby affecting their differentiation process. Additionally, it can activate osteoclast differentiation and lead to bone destruction [150]. Iron ions initiate synovial hyperplasia by regulating the expression of key genes (such as c-myc and mdm2), which are responsible for synovial cell proliferation and promote the occurrence and development of vascular synovitis [151]. Furthermore, ROS derived from NOX2 have been shown to inhibit antigen-dependent T cell responses and significantly reduce the severity of experimental arthritis in rats and mice [152]. In CD4 T cells, the lack of NOX2 induces Th17 cell production and reduces regulatory T cells in a ROS-dependent manner through the modulation of the transcription factors Foxp3 and RORγt [153]. Inhibition of the TRPM7 channel weakens ferroptosis in rheumatoid arthritis chondrocytes by suppressing the PKCα-NOX4 axis [154, 155]. A study found that decreased levels of the Nrf2 factor can lead to RA. Targeted activation of Nrf2 inhibits ROS production, thereby suppressing the proliferation and migration of fibroblast-like synovial cells, which resemble fibroblasts in rheumatoid arthritis [156]. Luo et al. found that RSL3 decreases Nrf2 and GPX4 in synovial cells [157]. Moreover, Nrf2 deficiency causes changes in the expression of SLC7A11, resulting in oxidative stress damage and exacerbating joint destruction [148]. An increased risk of RA may be associated with dysfunction in the antioxidant system of fibroblast-like synovial cells (FLS), and various strategies to inhibit FLS proliferation and restore synovial homeostasis hold promise as potential treatment directions [158]. In a recent study, it was discovered that fibroblast and other mesenchymal cells are highly sensitive to ferroptosis [159]. Researchers have found that the ferroptosis inducer, imidazole ketone erastin (IKE), reduces the number of synovial fibroblasts and alleviates synovial inflammation in a CIA mouse model. Some fibroblasts exhibit resistance to IKE-induced ferroptosis, and it has been observed that the tumor necrosis factor (TNF) transcription pathway is relatively more active in these cells. TNF, as a pro-inflammatory cytokine, promotes fibroblast activation. In synovial fibroblasts from RA patients, the addition of exogenous TNF can activate NF-κB and glutathione biogenesis, increasing resistance to IKE-induced ferroptosis in a dose-dependent manner. However, in fibroblasts without exogenous TNF, IKE treatment depletes glutathione. In the CIA mouse model, resistance of TNF-induced synovial fibroblasts to ferroptosis can be eliminated by adding a low dose of IKE (20 mg/kg, twice a week) and a low dose of TNF inhibitor. The combination of IKE and TNF inhibitor also increases the sensitivity of fibroblasts from RA patients to ferroptosis. Research has discovered that Semaphorin 5 A inhibits ferroptosis in rheumatoid arthritis by activating the PI3K-AKT-mTOR signaling pathway [160]. In terms of epigenetics [161], methylation mediated by SAM increases ferroptosis in rheumatoid arthritis by enhancing the GPX4 promoter in response to glycine. Antioxidant stress presents new mechanisms in mediating RA ferroptosis, where SIRT1 is transcriptionally suppressed by YY1 and inhibits ferroptosis in rheumatoid arthritis [162].

In terms of pharmacological interventions, it has been found that FDA-approved RA drugs such as sulfasalazine and indomethacin can inhibit cell growth and induce ferroptosis. The activity of sulfasalazine and indomethacin is largely reduced by ferroptosis inhibitors, such as ferrostatin-1, antioxidants, or the iron chelator DFO. DFO can inhibit ferroptosis by preventing iron ions from donating electrons to oxygen and generating ROS. Treatment with RSL3 (a ferroptosis inducer) has been found to downregulate SLC2A3 expression and induce ferroptosis in RA fibroblast-like synoviocytes (RA-FLS) [163]. Injection of ferrostatin-1 into the joints has been shown to increase collagen II expression, promote activation of the Nrf2 antioxidant system, and reduce cartilage degradation, which helps alleviate arthritis inflammation [156]. Some natural polyphenolic compounds can also significantly inhibit ferroptosis. Icariin can inhibit ferroptosis through the Xc-/GPX4 axis and enhance cell survival in LPS-induced synoviocytes [164]. Rhein sulfate [165] effectively controls CIA rat joint inflammation and improves joint bone erosion. It modulates the levels of ferroptosis-related signaling pathways, including ACSL4, SLC7A11, GPX4, and FTH1, and reduces the expression of MMP3 and MMP13, which could be one of the important mechanisms and pathways underlying its inhibitory effect on RA bone destruction. Curcumin has antioxidant and anti-inflammatory properties. It downregulates p53 and upregulates the expression of SLC7A11 and GPX4, suggesting that it improves cartilage degradation in osteoarthritis by inhibiting ferroptosis through the regulation of the p53 signaling pathway, indicating its potential therapeutic effect in osteoarthritis [166]. Additionally, Wan et al. found that baicalein protects cartilage by upregulating the AMPK/Nrf2/HO-1 signaling pathway and inhibiting ferroptosis in chondrocytes. It also reduces pain sensitivity associated with osteoarthritis and mitigates its progression [167]. Daji has effects such as dispersing cold, stopping pain, and reducing swelling. Its main active ingredient, total dajizin triterpenes, reduces the expression of ACSL4 in the rat model of RA, increases the expression of glutathione and GPX4, and upregulates Kelch-like ECH-associated protein 1 and HO-1 in the Nrf2/HO-1/GPX4 pathway, indicating that total dajizin triterpenes can inhibit cell aberrant ferroptosis by suppressing lipid peroxidation and thus exert therapeutic anti-RA effects [168]. Ge et al. found that sophoridine (SRI) improves cell proliferation, inflammatory cell infiltration, and bone destruction in the synovial tissue of the knee, ankle, and toe joints in a mouse model of RA. It also partially activates the expression of GSH, GPX4, and SLC7A11, and inhibits the expression of ROS and IL-18 [156]. Moreover, it was discovered that icariin may play a role in protecting synoviocytes from ferroptosis. Therefore, icariin can counteract the effects of RSL3 on iron content, lipid peroxidation, and relative proteins (SLC7A11, SLC3A2L, GPX4, TRF, NCOA4, and Nrf2) in synoviocytes [169]. It can be utilized as a novel therapeutic strategy for RA.

**Fig. 9:**
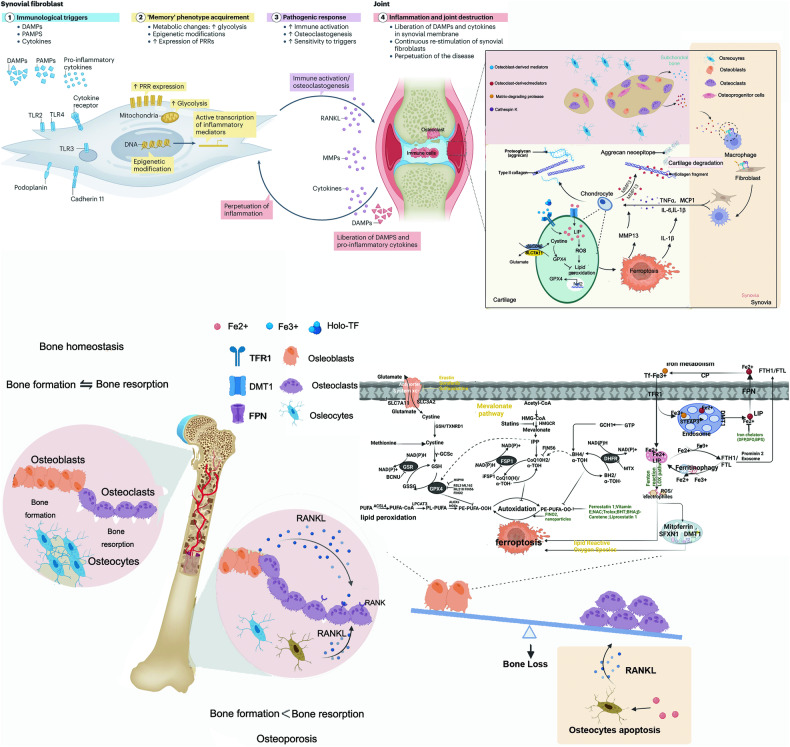
The mechanism of ferroptosis in RA. Ferroptosis is involved in the development of RA. DAMP damage-associated molecular pattern, PAMP pathogen-associated molecular patterns, RANKL receptor activator of nuclear factor-κ B ligand, MMP matrix metalloproteinases.

The above findings indicate the significant role of “ferroptosis” in the development of RA. Overall, ferroptosis acts as a triggering factor of inflammation, affecting the body’s immune regulatory system, promoting iron ion-induced lipid peroxidation, and inducing osteoclast differentiation while inhibiting osteoblast proliferation, leading to cartilage destruction and bone erosion. It is evident that glutathione peroxidase activity is reduced in polymorphonuclear leukocytes of RA patients. The ferroptosis inducer RSL3 can induce ferroptosis in synoviocytes and exacerbate synovial inflammation, leading to upregulation of transferrin receptor 1 (TFR1) and nuclear receptor coactivator 4 (NCOA4). Therefore, ferroptosis may serve as a novel therapeutic target for the prevention and treatment of RA. Improving RA can be achieved by inhibiting “ferroptosis” and preventing excessive lipid peroxidation due to the accumulation of free iron ions in cells, thereby providing a potential target for RA treatment (Fig. 9).

### Neurological autoimmune diseases (NAD)

NAD are characterized by inappropriate immune responses, including the presence of autoantibodies targeting self-antigens in the central nervous system and peripheral nervous system, leading to abnormal immune reactions and the occurrence of NAD. This group primarily encompasses a range of rare and complex conditions, including multiple sclerosis, autoimmune encephalitis, neuromyelitis optica spectrum disorders, autoimmune epilepsy, myasthenia gravis, and central nervous system demyelinating diseases, among others, and affects individuals across a wide age range [170]. The pathological features of non-neurological autoimmune neuropathies primarily involve immunopathological mechanisms associated with adaptive immune responses against antigens expressed in the nervous system, resulting in neuroinflammation. Neuroinflammation includes activation of resident microglial cells in the brain and neuronal stress and damage caused by infiltrating immune cells in the periphery [171]. Neurons, as long-lived terminally differentiated cells in the human body, experience numerous cellular stressors during the aging process, with oxidative stress being a crucial factor [172]. Oxidative stress arises from the excessive accumulation of intracellular reactive oxygen species (ROS). Excess ROS can cause DNA, protein, and lipid damage, leading to cell death [173]. The brain is particularly susceptible to oxidative stress due to its high oxygen demand, abundance of high-redox-active metals (such as iron and copper), and vulnerability of polyunsaturated fatty acids to oxidative damage, while the number of antioxidant factors is relatively limited [174, 175]. Ferroptosis is implicated as a mechanism underlying the loss of oligodendrocytes and demyelination in experimental autoimmune encephalomyelitis [176]. Tian et al. conducted a systematic investigation into the regulation of oxidative stress and the mechanisms by which human neurons cope with oxidative stress and unexpectedly discovered a neuron-specific ferroptosis pathway [177].

In the pathology of neuroinflammation, different types of glial cells can regulate neuronal iron deposition in a positive or negative manner, influencing the process of ferroptosis. Iron overload can activate microglia and astrocytes [178]. Inflammatory cytokines released by activated microglia and astrocytes (IL-1β, TNF-α, IL-6) upregulate the iron importer protein, DMT1, and downregulate the iron exporter protein, FPN1, promoting iron accumulation in neurons [179, 180]. IL-6 can also promote the release of ferritin from astrocytes, thereby preventing neuronal iron efflux mediated by FPN1 [181]. Furthermore, increased expression of heme oxygenase-1 (HO-1) in microglia and loss of copper chaperone protein (CP) activity in astrocytes contribute to iron deposition in the central nervous system (CNS) and neuronal death [182]. Oligodendrocytes, which contain the highest iron content in the CNS, may release a large amount of iron and damage neurons under pathological conditions such as demyelination [183]. Conversely, activated astrocytes can secrete BDNF and GDNF to mediate downregulation of DMT1, reducing iron accumulation in neurons [184]. Glial cells can also promote or inhibit ferroptosis by affecting neuronal oxidative stress levels. Activated microglia can release large amounts of reactive oxygen species (ROS), accelerating neuronal death [185].

### Multiple sclerosis (MS)

MS is an autoimmune disease characterized by inflammation and demyelination of the central nervous system, leading to progressive neurodegeneration and worsening of symptoms [186]. Recent studies have revealed abnormal iron metabolism in the brains of MS patients, which is closely associated with the occurrence and progression of MS [186]. Various factors contribute to oligodendrocyte and myelin damage, resulting in the release of stored iron into the extracellular space, which is taken up by microglia/macrophages. Iron uptake promotes M1 polarization of microglia/macrophages, contributing to inflammation progression and chronic active phase with slow expansion at the lesion site [187]. Ferroptosis has also been implicated in the progression of relapsing-remitting MS patients [188]. Studies have demonstrated that copper-induced ferroptosis mediates oligodendrocyte loss and demyelination. With disease progression, iron depletion in oligodendrocytes may impair myelin and, ultimately, axonal integrity in the white matter, which is believed to play a crucial role in chronic progressive MS [189].

Studies have demonstrated increased iron deposition and oxidative phospholipid levels in the brains of MS patients. Additionally, levels of GPX4, an important regulator of ferroptosis, are reduced in spinal cord neurons of both MS brains and experimental autoimmune encephalomyelitis (EAE) mice, indicating that ferroptosis is an early pathological event in EAE [190]. In the EAE model, elevated iron levels, lipid peroxidation, and mitochondrial morphological changes were observed in the spinal cord tissue of EAE mice, indicating the presence of classical biochemical alterations and pathological features of ferroptosis. The ferroptosis inhibitor Liproxstatin-1 (Lip-1) alleviated EAE development, improved motor function in mice, and prevented oligodendrocyte demyelination and neuronal death. Moreover, H&E staining combined with flow cytometry demonstrated that Lip-1 significantly reduced CD4 + T cell infiltration in the central nervous system, indicating that ferroptosis inhibition can ameliorate behavioral deficits, neuroinflammation, and neural damage in EAE [190]. ACSL4, a member of the long-chain ACSL family, activates polyunsaturated fatty acids (PUFAs) for their incorporation into negatively charged membrane phospholipids. These membrane-bound long-chain PUFAs are susceptible to oxidation, particularly under stressful conditions, subsequently triggering cell ferroptosis [191]. Previous research has shown that IFN-γ secreted by CD8 + T cells induces ferroptosis in tumor cells by regulating the expression of cystine/glutamate transporter (xCT), leading to lipid accumulation. In T cells, the coordination between fatty acids, IFN-γ, and ACSL4 mediates tumor ferroptosis and immune responses [110, 192]. The initiation and execution of ferroptosis are closely associated with intracellular lipid metabolism, and lipid-mediated ferroptosis may also play a role in T cell activation and related disease processes [193]. Further investigations revealed that ACSL4 mediates ferroptosis and neuroinflammation in EAE mice, enhancing T cell receptor (TCR) signaling and activating T cells, thereby participating in immune regulation and accelerating EAE progression. Inhibition of ferroptosis or genetic intervention targeting ACSL4 effectively alleviates EAE progression [190]. In terms of epigenetics, a recent study identified euchromatic histone-lysine N-methyltransferase 2 (EHMT2, also known as G9a), an inflammation-induced epigenetic regulator, as a candidate therapeutic target for neuroprotection [194]. In EAE mice and MS patients, neuroinflammation significantly induces G9a-mediated inhibitory dimethylation of histone H3 at lysine 9 (H3K9me2). Pharmacological inhibition of G9a activity demonstrates robust neuroprotection in in vitro and in vivo disease models. Mechanistically, G9a drives ferroptosis by epigenetically suppressing anti-ferroptosis genes. Thus, in central nervous system inflammation, ferroptosis plays a critical role in neuronal loss [194]. In the context of MS, ferroptosis at different stages was investigated, revealing increased ferroptosis during peak and progressive phases. NCOA4 expression was found to be increased during the peak and progressive phases of chronic maximum coping effort EAE, accompanied by an elevation in redox-active ferrous iron. These changes coincided with decreased antioxidant pathways (system xc-, glutathione peroxidase 4, and glutathione) and increased lipid peroxidation. Initiating ferroptosis inhibitor treatment two weeks after the peak paralysis phase of EAE in mice resulted in significant functional and pathological improvements. Autopsy samples from individuals with secondary progressive MS (SPMS) showed NCOA4 expression in macrophages and oligodendrocytes at the mixed active/non-active lesion edge, with iron ferritin-positive cells and iron-containing cells present in this region. The presence of fewer iron ferritin-expressing cells in areas with NCOA4 expression indicated iron ferritin degradation and the release of redox-active iron consistent with increased lipid peroxidation. These findings suggest a potential involvement of ferroptosis in the pathogenesis of EAE and SPMS [195]. The functionality of CD4 T cells in EAE can be exacerbated by inhibiting GPX4 [196, 197].

In the context of therapeutic interventions, van San et al. found several indications of ferroptosis in active and chronic lesions as well as cerebrospinal fluid of patients with multiple sclerosis (MS), reflected by elevated levels of (unstable) iron, oxidative phospholipids, and lipid degradation products [198]. In a preclinical model of relapsing-remitting MS, treatment with the candidate lead compound UAMC-3203, an inhibitor of ferroptosis, significantly delayed relapse and improved disease progression. Dimethyl fumarate, an FDA-approved treatment for MS, exerts neuroprotection by alleviating inflammation, oxidative stress, and ferroptosis through the NRF2/ARE/NF-κB signaling pathway, thereby ameliorating cognitive impairments in chronic cerebral hypoperfusion in rats [199]. Deferoxamine, a ferroptosis inhibitor, attenuates demyelination, promoting neuroprotection of the demyelinated optic nerve [200]. Extracellular vesicles derived from mesenchymal stem cells, specifically microRNA-367-3p, alleviate experimental autoimmune encephalomyelitis through targeting EZH2 and inhibiting ferroptosis in oligodendrocytes [201].

In summary, the findings confirm that ferroptosis is a detrimental and targetable factor in MS. These discoveries provide novel therapeutic options for patients with multiple sclerosis, complementing existing immunosuppressive strategies.

### Autoimmune diseases of the skin system

As the largest organ of the human body, the skin forms a mechanical and immune barrier against the environment. The immune system of the skin consists of cells from the innate immune system and cells from the adaptive immune system [202]. Signals from the innate immune system typically initiate immune responses in the skin, while cells and cytokines from the adaptive immune system sustain inflammation, ensuring effective defense against pathogens. However, this immune response can also lead to inflammatory skin diseases of autoimmune nature. The complexity of skin immune responses arises from extensive cross-talk between different cell types of the immune system, tissue cells, and pathogens [11, 203]. Common examples of autoimmune skin diseases include alopecia areata, psoriasis, vitiligo, skin dryness syndrome, scleroderma, pemphigus, and systemic lupus erythematosus [11]. In a study utilizing scRNA-seq data sets, the abnormal trends of genes related to ferroptosis and necroptosis were analyzed in several representative autoimmune diseases (psoriasis, atopic dermatitis, vitiligo, multiple sclerosis, and systemic sclerosis-associated interstitial lung disease). Furthermore, batch RNA-seq and qPCR were employed to evaluate cell line models and observed significant differences between normal and autoimmune disease samples involving ferroptosis. Ferroptosis was found to contribute to the imbalance of distinct keratinocyte lineages in psoriatic skin and the unique necroptosis-sensitive keratinocyte subpopulation in atopic dermatitis (AD) skin. The results also indicated the involvement of ferroptosis in the destruction of epidermal melanocytes in vitiligo. Abnormal ferroptosis has been detected in multiple sclerosis, systemic sclerosis-associated interstitial lung disease, Crohn’s disease, and autoimmune orchitis, and the cell line models used in this study identified pro-inflammatory factors that can drive changes in ferroptosis [204].

#### Psoriasis

Psoriasis is a common chronic inflammatory skin disease characterized by excessive proliferation of keratinocytes, immune cell infiltration, and accumulation of inflammatory cytokines [205]. Mao et al. identified three differentially expressed genes related to ferroptosis through bioinformatics analysis. SLC7A5, SLC7A11, and CHAC1 may influence the development of psoriasis by modulating ferroptosis [206]. Lipid metabolism abnormalities, particularly in the keratinocytes involved in psoriatic lesions, can be observed in patients with psoriasis. At the single-cell level, the lipid oxidation pathway in psoriatic keratinocytes is significantly upregulated compared to other cells such as fibroblasts, macrophages, dendritic cells, endothelial cells, and T cells, and it is highly associated with the Th22/Th17 pathway [206]. Cell death related to ferroptosis has also been activated in psoriatic skin lesions, as reported [22]. For instance, acyl-CoA synthetase long-chain family member 4 (ACSL4), prostaglandin-endoperoxide synthase 2 (PTGS2), and transferrin receptor (TFRC) are highly expressed in psoriatic skin lesions, while GPX4, ferritin light chain (FTL), and ferritin heavy chain 1 (FTH1) expression is lower compared to healthy skin lesions. Ferroptosis not only promotes cell death but also triggers inflammation in psoriatic keratinocytes. Research has shown that ferroptosis triggers and amplifies multiple inflammatory responses, enhances inflammation by releasing damage-associated molecular patterns, further activates immune cells, and significantly stimulates the expression of inflammatory cytokines, thus forming complex connections between inflammation and ferroptosis in psoriatic skin lesions [207]. Overexpression of ACSL4 in psoriasis enhances inflammation by activating ferroptosis [208]. In terms of iron metabolism, activation of ferroptosis due to iron overload may be a novel mechanism underlying the formation of skin lesions in psoriasis patients. Increased levels of lipid reactive oxygen species (ROS) and ferrous iron were observed in lesional epidermis of psoriasis vulgaris (PV) patients. Transmission electron microscopy confirmed the activation of ferroptosis in the epidermis of PV individuals, both in PV patients and in a psoriasis-like mouse model. Intradermal injection of the ferroptosis inducer RSL3 in psoriasis-like mice significantly promoted and aggravated psoriasiform dermatitis, and serum transferrin levels were elevated in PV samples. Furthermore, abnormal expression of certain iron metabolism-related genes was confirmed in the epidermis of PV cases, among which Cyb561d2 was found to promote ferrous iron overload and lipid peroxidation accumulation in HaCaT cells [209]. Additionally, Fer-1, an effective lipid peroxidation inhibitor, can inhibit ferroptosis-related changes in erastin-treated keratinocytes and block inflammation both in vitro and in vivo, reducing cytokine production, and alleviating psoriasiform dermatitis in the imiquimod-induced model [207]. However, how Fer-1 blocks the inflammatory response remains unclear, and identifying key pathogenic mediators in this process may provide new and more specific therapeutic targets for psoriasis.

#### Atopic dermatitis, vitiligo, systemic sclerosis-related interstitial lung disease

Vitiligo is an autoimmune skin disorder with a global prevalence of 1–2%. Patients with vitiligo experience depigmentation due to the autoimmune attack of melanocytes by T cells, resulting in the formation of white patches on the skin. This disease significantly impacts the physical appearance of patients, affecting their social confidence and imposing psychological stress [210]. Increasing evidence has revealed the crucial role of IFN-γ-CXCL9/10-CXCR3 pathway-related proteins in epidermal recruitment and CD8 + T lymphocyte-mediated melanocyte destruction during the progression of vitiligo [211–213]. Among various hypotheses proposed for the pathogenesis of vitiligo, the oxidative stress-induced immune response leading to melanocyte death remains widely accepted. Elevated levels of ROS due to oxidative stress can disrupt molecular and organelle functions, triggering further immune responses and eventually resulting in melanocyte apoptosis [214]. In the skin, melanocytes possess higher levels of biologically available iron compared to keratinocytes. Under external oxidative stimulation, increased oxidation of unsaturated fatty acids occurs concurrently with Fe2+ in melanocytes, significantly enhancing their susceptibility to ferroptosis [215]. Studies have shown iron deficiency in the blood of vitiligo patients. Erastin reduces cell viability, leading to oxidative stress, iron overload, and accumulation of lipid peroxides in human epidermal melanocytes. Erastin induces the expression changes in ferroptosis markers and inhibits melanin synthesis in melanocytes, which can be attenuated by N-acetyl-L-cysteine (NAC) pretreatment or post-treatment in vitro. Overall, ferroptosis may play a role in the progression of vitiligo. Erastin can induce ferroptosis in human epidermal melanocytes, while NAC can protect melanocytes from ferroptosis in vitro [216]. Another study found that IFN-γ downregulates melanin regulators (DCT, KITLG, FZD5), leading to decreased melanin production in melanocytes, and promotes lipid peroxidation (ROS) and ferroptosis in primary melanocytes derived from patients. However, treatment with the fully human monoclonal IFN-γ antibody EI-001 (experimental code 2A6Q) at a concentration of 10 μg/ml alleviates the inhibitory effect of IFN-γ on melanocyte viability, increases melanin production, reverses IFN-γ-induced ROS accumulation and ferroptosis, and significantly reduces melanocyte apoptosis as detected by flow cytometry. This study provides strong evidence that treatment with the fully human monoclonal IFN-γ antibody EI-001 alone effectively inhibits IFN-γ-induced melanocyte damage, restores melanin production, and holds great potential for vitiligo therapy [217]. In terms of pharmacological intervention, it has been found that treatment with baicalein significantly alleviates RSL3-induced damage. Additionally, baicalein upregulates GPX1 and reduces TFRC levels in melanocytes treated with RSL4 + FAC. By upregulating GPX4, baicalein protects melanocytes from ferroptosis [218, 219]. Ferroptosis may be widely present in the occurrence and development of vitiligo and could be proposed as a potential therapeutic target.

#### Myositis/Dermatomyositis

Idiopathic inflammatory myositis (IIM) is an inflammatory disease characterized primarily by muscle weakness, and the underlying pathological mechanisms are still unclear. The classification proposed by Bohan/Peter in 1975 categorizes IIM into dermatomyositis (DM), polymyositis (PM), and inclusion-body myositis [220]. Clinical manifestations of IIM include muscle weakness, myalgia, characteristic skin rashes, elevated serum creatine kinase (CK) levels, muscle fiber degeneration and regeneration, chronic mononuclear cell infiltration, and perifascicular atrophy in muscle biopsies [220]. The exact mechanisms of IIM pathogenesis remain unknown, but it is widely believed to result from the interplay of genetic, environmental, immune, and non-immune factors (endoplasmic reticulum stress, oxidative stress, abnormal autophagy regulation, hypoxia, and angiogenesis) [221]. IMNM, currently considered the most common type of IIM, may occur following viral infections. It is characterized by severe acute or subacute proximal muscle weakness, typically presenting with elevated CK levels, minimal inflammatory infiltrates, and significant muscle necrosis in muscle biopsies [222]. Ferritin serves as a major intracellular iron storage, and its increased levels indicate iron accumulation in muscle inflammation patients [223, 224]. Several population-based studies focusing on PM/DM have found an association between the severity and prognosis of PM/DM and hyperferritinemia, along with its complications such as interstitial lung disease [225, 226]. Additionally, mitochondrial dysfunction, as a hallmark event that terminates ferroptosis and leads to ROS accumulation, has previously been reported as a critical pathogenic mechanism in IIM [227, 228]. Ma et al. found that p53 may contribute to the pathogenesis of IIM by upregulating the expression of SAT1 and subsequently inducing the overexpression of ALOX15. The overexpression of p53, SAT1, ALOX15 proteins, and iron accumulation may lead to ferroptosis in cells, and higher expression levels of p53, SAT1, and ALOX15 proteins could be associated with more severe muscle inflammatory lesions and increased muscle damage [229]. Deng et al. suggested that ROS mediated by NOX2 and NOX4 in NADPH oxidases may participate in the pathogenesis of IIM through the ferroptosis pathway. They proposed that NOX2 and NOX4 play a crucial role in the development of IIM, possibly by depleting NADPH to generate excessive ROS, leading to oxidative damage to tissue cells and inducing ferroptosis. NADPH, NOX2, NOX4, and ROS have the potential to serve as new indicators for evaluating the extent of muscle tissue damage in IIM [230]. In terms of the oxidative stress-induced ferroptosis pathway, Liu et al. found that the Nrf2/ARE pathway can inhibit the inflammatory infiltration of macrophages in autoimmune myositis rats [231].

Vitamin E and selenium are important antioxidants that can prevent lipid peroxidation and exhibit a protective effect against ferroptosis [232–234]. It has been found that patients with chronic malabsorption and selective IgA deficiency, who are deficient in vitamin E and selenium, develop PM when treated with iron dextran, which is believed to be related to lipid peroxidation induced by free radicals activated by free iron [235]. Therefore, it can be observed that ferroptosis is involved in the occurrence and progression of IIM, and further exploration is needed to elucidate the exact role of ferroptosis in IIM.

#### Systemic sclerosis (SSc)

SSc is an autoimmune disease characterized by localized or diffuse skin thickening and fibrosis. It is associated with vascular abnormalities, skin and organ fibrosis, immune dysfunction, and excessive deposition of extracellular matrix, affecting multiple organs including the skin, lungs, stomach, and kidneys. SSc has a worldwide distribution, with an average onset age between 30 and 50, and a higher prevalence in females than males [236]. The pathogenesis of SSc remains unclear, but it is believed to be influenced by genetic and environmental factors. Immune dysfunction, vascular damage, multiple organ fibrosis, and their interactions are the main factors contributing to the development of SSc [24]. Recent studies have demonstrated the presence of ferroptosis in skin and lung tissues of SSc mice with enhanced ACSL4 expression. Inhibiting ACSL4 effectively prevents fibrosis progression and provides protection against the inflammatory environment. Additionally, a positive regulatory relationship between LPS-induced macrophage activity and ferroptosis sensitivity has been observed. Knocking down calpain reduces both the sensitivity of inflammatory macrophages to ferroptosis and ACSL4 expression, while its overexpression leads to ACSL4 activation. Furthermore, pharmacological inhibition of calpain reduces ferroptosis and fibrotic capacity in mice [237]. In terms of biomarkers, studies have found that ferroptosis is upregulated in SSc patients and is involved in the modulation of cell proliferation, differentiation, and migration. IL-6 and CYBB are key ferroptosis-related genes in SSc. Ferroptosis and related genes may serve as promising targets for the treatment of SSc [238]. Pulmonary fibrosis (PF), as the end-stage clinical phenotype of systemic sclerosis-associated interstitial lung disease (ILD), is often triggered by alveolar injury, and ferroptosis has been identified as a key aggravating factor in this disease. UHRF1 mediates ferroptosis in type II alveolar epithelial cells (AEC2) through the epigenetic repression of GPX4 and FSP1 genes [239]. In terms of intervention, a study found that curcumin oil alleviates bleomycin-induced pulmonary fibrosis in mice by inhibiting ferroptosis [240]. Moreover, it is important for future researchers to focus on the mechanisms of ferroptosis in systemic sclerosis-associated interstitial lung disease.

### Autoimmune disease hepatitis

Autoimmune liver diseases are common clinical conditions, encompassing autoimmune hepatitis (AIH), primary biliary cholangitis (PBC), and primary sclerosing cholangitis (PSC). These diseases are primarily characterized by immune-mediated inflammatory liver lesions. The pathogenesis involves both innate and adaptive immune responses targeting bile duct cells and various extrahepatic tissues [241–243].

AIH is an immune-mediated chronic progressive liver disease. As a prototypical autoimmune disorder, AIH can occur at any age and in any ethnic group, with a female predominance in a ratio of 4:1 compared to males [244]. The histological features of the disease include moderate to severe interface hepatitis and lymphocyte infiltration, while serum markers consist of the presence of self-antibodies and elevated levels of IgG [245]. The pathogenesis of AIH is characterized by T lymphocyte activation, release of pro-inflammatory cytokines, infiltration, and destruction of hepatic parenchyma, resulting in sustained immune-mediated liver injury and functional impairment [246].

Liver cell injury plays a central role in the progression of AIH [246]. Guang et al. has demonstrated the involvement of ferroptosis in Con A-induced liver cell injury, accompanied by increased ROS generation, accumulation of labile iron, and elevated levels of lipid peroxidation marker MDA. Additionally, they found a significant reduction in GPX4 and xCT proteins in mice treated with Con A. Pre-treatment with the ferroptosis inhibitor fer-1 significantly attenuated liver cell injury in AIH. Furthermore, in Con A-treated mice, the expression of caveolin-1 (Cav-1) was prominently suppressed, while fer-1, the ferroptosis inhibitor, could rescue Cav-1 expression and inhibit the generation of reactive nitrogen species (RNS). Therefore, the study suggests that RNS-mediated ferroptosis downstream of Cav-1 is a crucial step driving acute immune-mediated liver injury [247]. Ting Zeng et al. investigated ferroptosis-related markers in the Con A-induced AIH mouse model and identified the crucial role of indoleamine 2,3-dioxygenase 1 (IDO1)-dependent ferroptosis and RNS in the AIH mouse model. IDO1 is an intracellular heme enzyme induced by pro-inflammatory cytokines, such as interferon-gamma (IFN-γ), and other immune mediators, which helps to suppress T cells and NK cells. Previous studies have shown that IDO1 is typically expressed in inflammatory cells and tissues, promoting an increase in NO and hydrogen peroxide. However, studies have not reported on whether IDO1-mediated immunosuppression plays a key regulatory role in autoimmune diseases. The authors reported that IDO1 induces ferroptosis by exacerbating nitrosative stress, promoting ferritin degradation, and accumulation of labile iron. Therefore, understanding the molecular mechanisms and signaling pathways of ferroptosis may provide new diagnostic and therapeutic approaches for regulating liver cell survival and death in AIH [248]. Jiang et al. found a simultaneous occurrence of ferroptosis in liver cells and a protective increase in FGF4 during Con A-induced AIH liver injury. In addition, liver-specific FGF4 knockout mice were more prone to accumulate lipid peroxides and free iron, leading to more severe liver damage and inflammation. Conversely, treatment with non-mitogenic recombinant FGF4 (rFGF4) alleviated liver injury and liver cell ferroptosis. This study suggests that FGF4 can play an immunoregulatory role in the progression of AIH [249]. In the liver-specific antigen S100-induced AIH mouse model, Zhu et al. observed an upregulation of cyclooxygenase 2 (COX2) and ACSL4, and downregulation of GPX4 and ferritin heavy chain 1 (FTH1) levels. Supplementation with ferrostatin-1 (fer-1) restored the aforementioned phenotype. Furthermore, when adenovirus-mediated silencing of hepatic GPX4 expression was employed, COX2 and ACSL4 levels were significantly upregulated in the AIH model mice, exacerbating liver injury. The authors also demonstrated the critical role of the Nrf2/HO-1 signaling pathway in inhibiting ferroptosis. These results contribute to our understanding of molecular interactions in AIH and provide directions for developing new therapeutic strategies [250]. Yang Liu et al. identified the dysregulated miRNAs in the Con A-induced AIH mouse model using microarray chips. A total of 49 miRNAs were screened (31 upregulated and 18 downregulated), resulting in the prediction of 959 target genes. These target genes were annotated in 47 signaling pathways, including “Wnt signaling pathway,” “Hippo signaling pathway,” “ferroptosis pathway,” and “mitogen-activated protein kinase (MAPK) signaling pathway,” suggesting that ferroptosis may serve as a potential novel target for AIH therapy [248]. A study revealed the preventive role of FGF4 in the progression of AIH. Treatment with rFGF4 inhibits ferroptosis in liver cells by increasing CISD3 levels and activating the Nrf2/HO-1 signaling pathway [251].

In summary, it is evident that ferroptosis, as a new form of cell death, is involved in the development and progression of liver inflammation, fibrosis, and carcinogenesis. It may become an important target for the treatment of chronic liver diseases. However, its translation into clinical therapeutic strategies still requires time. Future research should address the following challenges: 1) the interaction between abnormal cytokines and autoantibodies in AIH patients and key regulatory proteins of ferroptosis needs to be clarified to determine core regulatory targets; 2) it is unclear whether ferroptosis inhibitors exert their effects by inhibiting liver cell ferroptosis or by impacting immune regulatory cell ferroptosis; 3) whether immune cells also modulate the occurrence and progression of AIH through the regulation of ferroptosis remains to be investigated. Therefore, further investigation into the key role of ferroptosis in AIH and elucidation of the molecular mechanisms of this novel form of cell death are necessary. This will allow for the precise control of disease progression at the cellular and organelle levels while minimizing damage to surrounding normal tissues.

### Sjogren’s syndrome (SS)

SS is a chronic inflammatory autoimmune disease characterized by lymphocyte proliferation and progressive exocrine gland damage. Clinical manifestations include impaired salivary and lacrimal gland function, as well as systemic involvement of multiple organs. Autoantibodies and hypergammaglobulinemia can be detected in the serum [252]. SS is classified into two types based on the presence or absence of other connective tissue diseases: secondary SS and primary SS (pSS), with the former commonly associated with systemic lupus erythematosus, rheumatoid arthritis, etc. pSS is a global disease with an estimated prevalence of 0.3%-0.7% in China, predominantly affecting females. The male-to-female ratio ranges from 1:9 to 1:20, and the onset age is typically between 40 and 50, although it can also occur in children [253]. The exact etiology and pathogenesis of pSS remain unclear, with current evidence suggesting a combination of genetic factors, viral infections, and hormonal abnormalities leading to immune dysfunction [254]. A study identified that a combination of six ferroptosis-related genes, including TBK1, SLC1A4, PIK3CA, ENO3, EGR1, and ATG5, could serve as optimal markers for diagnosing pSS. The analysis of these six genes in combination accurately diagnosed pSS occurrence [255]. Furthermore, Peng et al. [256]. utilized proteomic analysis to examine plasma exosomes from pSS patients, revealing the presence of 24 differentially expressed proteins (DEPs). These proteins are involved in primary biological processes and signaling pathways associated with ferroptosis. Notably, copper-binding protein (CP) and transferrin (TF) were representative among the enriched DEPs. Additional findings include the presence of epithelial cell-derived proteins associated with ferroptosis in plasma exosomes from pSS patients, the potential involvement of complement C5 and C9 in ferroptosis, and variations in the levels of ferroptosis-related proteins in the exosomes being more reflective of epithelial cell pathology compared to plasma. Increased levels of IFN-γ in SS lead to salivary gland epithelial cell (SGEC) death. In a recent study [257], ferroptosis was found to play a role in SS-related SGEC death and SS pathogenesis. Inducing ferroptosis or treating with IFN-γ in Institute of Cancer Research (ICR) mice exacerbates the symptoms of SS, while inhibition of ferroptosis or the IFN-γ signaling pathway in non-obese diabetic (NOD) mice models alleviates both ferroptosis in the salivary gland and SS symptoms. IFN-γ activates STAT1 phosphorylation and downregulates solute carrier family 3 member 2 (SLC3A2), glutathione, and GPX4, thereby triggering ferroptosis in SGEC. Inhibition of JAK or STAT1 in SGEC rescues the downregulation of SLC3A2 and GPX4 induced by IFN-γ, as well as IFN-γ-induced cell death.

In terms of interventions [258], treatment with exosomes derived from stem cells obtained from human exfoliated deciduous teeth (SHED-exos) promotes salivary flow rate in NOD mice while reducing caspase-3 levels and the number of apoptotic cells in the submandibular glands (SMGs). SHED-exos inhibit the expression of markers associated with damage to the glands, such as autophagy, pyroptosis, NETosis, ferroptosis, necroptosis, and oxidative apoptosis. Overall, SHED-exos suppress epithelial cell death, which is responsible for promoting salivary secretion. The inhibition of inflammation-induced epithelial cell apoptosis by SHED-exos is correlated with the suppression of p-ERK1/2 activation.

### Inflammatory bowel disease (IBD)

IBD refers to a group of chronic nonspecific gastrointestinal inflammatory disorders, including Crohn’s disease (CD) and ulcerative colitis (UC) [259, 260]. As a complex disease involving multiple factors and genetic interactions, the etiology and pathogenesis of IBD remain unclear, but it is generally believed to be associated with environmental factors, genetic susceptibility, gut microbiota, and immune responses [260]. Ferroptosis plays an important role in the pathogenesis of IBD intestinal mucosal barrier [261]. Research has found morphological changes in ferroptosis cells, such as mitochondrial shrinkage and reduced mitochondrial cristae, in the intestinal epithelial cells of UC patients and experimental colitis mice. It has also been observed that the ferroptosis biomarker prostaglandin G/H synthase 2 (PTGS2) is increased in the intestinal epithelial cells [262]. Markers of ROS, COX2, and ACSL4 are highly expressed at tissue, mRNA, and protein levels [263–265], while superoxide dismutase (SOD), an enzyme that inhibits ROS production, is downregulated [265]. As previously mentioned, iron accumulation, glutathione (GSH) depletion, GPx4 inactivation, and lipid peroxidation (LPO) are key features of ferroptosis, and these characteristics are also observed in the intestinal epithelial cells of IBD patients and mice. Iron overload can induce colitis by modulating ferroptosis and disrupting the gut microbiota [266]. Ferroptosis mediated by ACSF2 is associated with ulcerative colitis [267]. SLC6A14 promotes epithelial cell ferroptosis through the C/EBPβ-PAK6 axis in ulcerative colitis [268]. The metabolic pathways of ferroptosis are divided into exogenous (transporter-dependent pathways) and endogenous (enzyme-regulated pathways). Ferroptosis occurrence is influenced by the exogenous pathway (iron metabolism, amino acid-GSH/GPx4) and the endogenous pathway (endoplasmic reticulum stress, Nrf2/HO-1 signaling pathway, AKT/IKK/P65, and ERK/IKK/P65 signaling pathway cascades), thereby regulating IBD. In terms of iron metabolism, studies have found increased iron content in IBD intestinal tissues, higher levels of Fe2+ involved in the Fenton reaction of cellular ferroptosis, and significantly elevated mRNA and protein levels of FTL and FTH1. FTH1-positive signals are mainly observed in the intestinal epithelial cells, indicating that ferroptosis mainly occurs in epithelial cells. Additionally, DFO reduces ferroptosis and counteracts colitis by chelating excess free iron [262]. Hereditary hemochromatosis, characterized by a recessive mutation in the hemochromatosis (Hfe) gene, has been shown to elevate malondialdehyde (MDA) levels in colonic tissues of Hfe gene knockout mice, suggesting that iron overload promotes oxidative damage to intestinal cells. Furthermore, colonic mucosal damage in mice is associated with increased susceptibility to colitis, indicating the significant role of iron overload in the pathogenesis of colitis. Iron overload in the intestines leads to ROS accumulation and cell ferroptosis, which may be a pathogenic mechanism in colitis [269]. Iron deficiency is a common cause of anemia in IBD patients, and oral iron supplements are commonly used in clinical management of iron-deficiency anemia [270]. However, animal studies have shown that oral iron supplementation can alter the composition and metabolism of gut microbiota, worsening intestinal inflammation [271, 272]. A clinical study found that as the intake of iron supplements increased (categorized as low, moderate, and high iron dose groups with intakes of 2.99 and 3.6 mg/4,184 kJ), the odds ratio for developing UC increased, indicating an increased disease risk with higher iron intake [273]. Inappropriate iron supplementation or excessive intake of iron supplements often leads to ROS accumulation through the Fenton and Haber-Weiss reactions, triggering oxidative stress, LPO, damage to intestinal epithelial cells, and even cell death, which disrupts the intestinal mucosal barrier function. Therefore, intravenous iron supplementation is recommended as the initial treatment for clinically active UC, severe anemia, and patients who are intolerant to oral iron. Glycyrrhizin, known for its antioxidant and anti-inflammatory activities, holds promise as an effective drug for IBD [274]. Recent studies have found that supplementing glycyrrhizin in colitis mice upregulates ferritin expression, increases cellular iron storage, lowers cellular iron levels, and further inhibits ferroptosis in the colitis model epithelial cells [275]. Currently, there is limited research on how iron overload exacerbates intestinal mucosal damage and inflammation through ferroptosis. Exploring the relationship between iron metabolism and IBD, as well as related targets, may provide new directions and insights for modulating cellular ferroptosis and alleviating intestinal mucosal damage.

In terms of the GSH/GPx4 pathway, both UC and CD patients show reduced GPx4 activity in the intestinal epithelial cells during active disease, suggesting a close association between IBD and ferroptosis [262, 276]. Further research has found that in intestinal epithelial cells with reduced or deficient GPx4, ACSL4 induces the release of interleukin 6 (IL6) and chemokine (C-X-C motif) ligand 1 (CXCL1) by regulating PUFA, especially Aa, leading to inflammation. Additionally, ACSL4 restricts the production of anti-inflammatory Aa metabolites (such as epoxyeicosatrienoic acids, EETs). Animal studies have shown that mice lacking GPx4 are more susceptible to colitis compared to wild-type mice, highlighting the important role of GPx4 in protecting the intestine from LPO damage and maintaining intestinal homeostasis [276]. Recent studies have found that Pannexin plays a significant role in this process [277]. It has been found that curculigoside improves the sensitivity of intestinal epithelial cell GPx4 to selenium, promotes GPx4 expression, and alleviates histological damage of the colon in UC mice induced by dextran sulfate sodium (DSS) [278]. Similarly, clinical studies have shown that appropriate selenium supplementation in selenium-deficient individuals enhances GPx4 activity, prevents cell ferroptosis, and thus prevents the occurrence of IBD [278]. The ferroptosis inhibitor Liproxstatin-1 (Lip-1) enhances the expression of the anti-ferroptosis system by inhibiting LPO, increasing GSH and FSP1 concentrations, and restoring GPx4 to normal levels [279]. It has also been demonstrated to improve symptoms in colitis patients and DSS-induced colitis in mice [264]. Recent studies have found that the traditional Chinese medicine formula Shao Yao Tang, by activating GPx4, inhibits ferroptosis in colonic epithelial cells, alleviates colitis, inhibits inflammation, and restores intestinal barrier function, providing a scientific basis for the clinical efficacy of Chinese medicine formulas in treating IBD [280]. Moreover, increasing evidence suggests that Nrf2 is involved in the occurrence of ferroptosis and can regulate the expression of antioxidant response elements such as GPx4 [281]. In intestinal epithelial cells of DSS-induced colitis mice, downregulation of the Nrf2-GPx4 signaling pathway promotes ferroptosis, while Furin protease can inhibit ferroptosis and protect intestinal epithelial cells by activating the Nrf2-GPx4 signaling pathway [282, 283]. It has been found that sulfasalazine inhibits cellular ferroptosis by suppressing the activity of the heterodimeric xc- that transports GSH precursors [284]. Sulfasalazine is a commonly used drug in the clinical treatment of IBD, and it exerts its anti-inflammatory effects by affecting the synthesis of prostaglandins. Further investigation of the concentration of sulfasalazine and its effects on intestinal epithelial cells in IBD, balancing the relationship between inflammation inhibition and ferroptosis, may contribute to improving the efficacy of the drug through animal and clinical experiments.

Research suggests that endoplasmic reticulum (ER) stress not only promotes the development of UC but is also involved in the occurrence of ferroptosis [285]. Protein kinase R-like ER kinase (PERK) is the main sensor of ER stress. RSL3 is an inhibitor of GPx4, and studies have found that the PERK inhibitor GSK414 not only suppresses the expression of the ER stress signaling pathway eIF2α/ATF4/CHOP induced by RSL3 but also reduces cellular ferroptosis, improving experimental colitis in mice. This indicates that ferroptosis regulates UC through ER stress-mediated intestinal epithelial cell death [262]. Further research has found that the interaction between phosphorylated nuclear factor κB (NF-κB) p65 and its regulator eIF2α inhibits ER stress-mediated intestinal epithelial cell ferroptosis, suggesting that NF-κB p65 may be a potential therapeutic target for UC [286].

In terms of the Nrf2/HO-1 signaling pathway, Nrf2 not only inhibits ferroptosis and protects intestinal epithelial cells through the Nrf2-GPx4 signaling pathway but also promotes ferroptosis through the Nrf2/HO-1 pathway. On the one hand, Nrf2 and HO-1 are significantly upregulated in mouse colitis, exerting anti-inflammatory and antioxidant effects [264, 287]. Astragalus polysaccharide (APS) can prevent experimental colitis in mice and ferroptosis in human Caco-2 cells by inhibiting this signaling pathway [155], suggesting that ferroptosis may be regulated by the Nrf2/HO-1 signaling pathway in DSS-induced UC. On the other hand, the excessive activation of Nrf2/HO-1 disrupts the balance of iron ion metabolism, leading to ferroptosis [288, 289]. Ferroptosis suppressor protein 1 (Fer1) downregulates the expression of Nrf2/HO-1, chelates unstable Fe2+ in the iron pool, decreases free iron concentration, and inhibits ferroptosis, improving symptoms in colitis patients and DSS-induced colitis in mice [264, 290]. The specific mechanism of the Nrf2/HO-1 signaling pathway in regulating ferroptosis is still unclear and requires further investigation.

In the AKT/IKK/P65 and ERK/IKK/P65 signaling pathways, studies have found that the expression of MELK is elevated in colitis patients and mouse models. The MELK inhibitor OTSSP167 protects intestinal epithelial cells by maintaining a normal composition of gut microbiota and balancing gut microbiota distribution, inhibiting ferroptosis, reducing the expression of pro-inflammatory factors in intestinal tissues, and suppressing the AKT/IKK/P65 and ERK/IKK/P65 signaling cascades in vitro and in vivo, thereby exerting a protective effect on the intestinal tissues of colitis mice [291, 292]. Ferroptosis may be regulated in IBD intestinal epithelial cells by phosphorylated AKT, ERK, IKK, and P65, providing new perspectives and strategies for the treatment of IBD, with MELK potentially being an effective target for IBD therapy. Previous studies have proposed a correlation between increased incidence of IBD and PUFA (such as Aa) intake in the diet [293]. Large-scale prospective clinical trials conducted in CD patients have found that supplementation with PUFA may worsen IBD symptoms, indicating disrupted gut homeostasis [294]. α-tocopherol, a potent active hydrolysis product of vitamin E, can prevent PUFA-induced lipid peroxidation, cytokine production, and neutrophil infiltration, to some extent, inhibiting cell ferroptosis [276]. Additionally, direct supplementation of monounsaturated fatty acids (MUFAs) in the diet to replace PUFA, which is prone to lipid peroxidation on cell membranes, can prevent the accumulation of lipid ROS and the occurrence of ferroptosis [295]. Moreover, CoQ10H2, a lipophilic free radical scavenger, can counteract lipid ROS, thereby inhibiting ferroptosis. A recent randomized controlled trial found that CoQ10 supplementation, which is reduced to CoQ10H2 under the action of FSP1, effectively alleviates inflammation in mild to moderate UC patients during remission [296]. At present, there is limited direct clinical research on the relationship between ferroptosis and IBD, but a series of studies have proposed a hypothesis that a more balanced diet (with balanced iron, selenium, CoQ10, and fatty acids) may be a better choice for improving IBD symptoms, maintaining gastrointestinal health, and preventing the occurrence of IBD. These viewpoints require extensive animal experiments and clinical studies to confirm.

In terms of interventions, Delarose mitigates DSS-induced colitis in mice by inhibiting ferroptosis and improving gut microbiota composition [297]. A high-fat diet alleviates colitis by inhibiting ferroptosis through the solute carrier SLC7A11 [298]. Inhibition of aryl hydrocarbon receptor prevents oxidative stress and ferroptosis in intestinal epithelial lymphocytes [299]. AMPK activation relieves sodium dextran sulfate-induced colitis by inhibiting ferroptosis [300]. In terms of antioxidant stress, α-lipoic acid alleviates sodium dextran sulfate-induced ulcerative colitis by regulating the Keap1-Nrf2 signaling pathway and inhibiting ferroptosis [301]. In the field of biological therapies, human umbilical cord-derived mesenchymal stem cell-derived exosomes shuttle miR-129-5p to mitigate inflammatory bowel diseases by inhibiting ferroptosis. Xue-Jie-San restricts Crohn’s disease ferroptosis by inhibiting the FGL1/NF-κB/STAT3 positive feedback loop [302]. MSC therapy improves the severity of DSS-induced colitis by modulating gut microbiota, immune responses, and the ferroptosis pathway [303]. In the realm of natural compounds, ellagic acid alleviates glucosamine-sulfate-induced ulcerative colitis in mice by modulating gut microbiota and inhibiting ferroptosis [304]. Rehmannioside attenuates sodium dextran sulfate-induced colitis in mice by modulating gut microbiota and inhibiting ferroptosis [305]. Kumatakenin suppresses ferroptosis in colitis mouse epithelial cells by regulating the Eno3-IRP1 axis [306]. β-caryophyllene serves as an inhibitor of ferroptosis, improving experimental colitis [274]. Inhibition of Prdx6-induced ferroptosis in epithelial cells contributes to the alleviation of colitis by glycyrrhizin [307].

### Ocular autoimmune diseases

Ocular autoimmune diseases refer to conditions where the body loses tolerance to self-antigens, leading the immune system to attack ocular tissues causing damage [308]. Common triggers for ocular autoimmune diseases include genetic, environmental, and gender factors. Examples of these diseases include uveitis, glaucoma, thyroid eye disease, sympathetic ophthalmia, immune keratitis, dry eye syndrome, optic neuritis, and rheumatologic associated dry eye [309]. The cornea of the eye harbors a rich array of immune cells and nerve fibers, making it susceptible to characteristic changes induced by systemic autoimmune and vasculitic diseases, resulting in corneal manifestations like immune cell infiltration, alterations in nerve fibers leading to changes in corneal mucosal tissues, decreased tear secretion, modifications in meibomian gland structure and function manifesting as symptoms and signs of dry eye syndrome, posing challenges in treatment [310]. Recent research highlights the critical role of iron in ocular inflammation by promoting the generation of reactive oxygen species, wherein intraocular iron injections trigger oxidative stress, subsequent development of geographic atrophy, and sympathetic ophthalmia [311]. Currently, ferroptosis has been found to be closely associated with various biological conditions of autoimmune-related ocular diseases [312].

#### Uveitis

Uveitis is a complex ocular inflammatory disease that, without timely and thorough pharmacological treatment, can severely impact vision due to recurrences and associated complications [313]. Oxidative stress plays a crucial role in the pathogenesis of uveitis; during peak inflammation, excessive expression of iNOS leads to uncontrolled elevation of NO levels, disrupting the blood-aqueous barrier, exacerbating inflammation. The excess production of ROS triggers lipid peroxidation in iris epithelial cell membranes and photoreceptor mitochondria, resulting in inflammation cell chemotaxis and irreversible retinal damage [314, 315]. Ferroptosis, induced by lipid peroxidation, is dependent on metabolites ROS, phospholipids containing polyunsaturated fatty acid chains (PUFA-PL), and the transition metal iron. Intracellular and extracellular signals and environmental stress can modulate ferroptosis by regulating cell metabolism and ROS levels [316]. ROS and lipid ROS play pivotal roles in ferroptosis; studies report increased SOD can inhibit ROS levels. Intracellularly, iron accumulation inhibits antioxidant systems; iron can directly generate excess ROS via Fenton reaction, leading to oxidative damage [317]. Recent research indicates that iron chelation is a widely safe and effective strategy for treating uveitis by inhibiting ferroptosis. Arora et al. found that DIBI, a polymer hydroxypyridinone iron chelator, reduces ocular inflammation in endotoxin-induced uveitis by decreasing local and systemic endotoxin-induced ocular inflammation. Treatment with DIBI in endotoxin-induced uveitis (EIU) reduced leukocyte activation and improved functional capillary density (FCD) of iris microcirculation [318]. Toxicity studies indicate acute and chronic administration of DIBI has no adverse effects on the eyes. In a local EIU model, DIBI demonstrated reduced leukocyte activation and restoration of FCD/DCD ratio, providing evidence for its anti-inflammatory properties. Regarding iron metabolism, the iron chelator deferoxamine mesylate can treat experimental uveitis in Lewis rats, resulting in a significant reduction in choroidal inflammation and retinal damage [319]. These findings suggest that in experimental uveitis, the severity of ocular inflammation and tissue damage may be mediated by hydroxyl radicals catalyzed by iron, thus deferoxamine mesylate can serve as an anti-inflammatory agent.

#### Glaucoma

Glaucoma is the leading global irreversible blinding eye disease, affecting individuals of all age groups. Clinically manifested as progressive visual field defects and optic nerve damage, the selective and irreversible loss of retinal ganglion cells (RGCs), the central input neurons of the retina, forms the basis of glaucomatous pathology [320]. Recent research has revealed that glaucoma is essentially an autoimmune disease, with the patient’s own T cells being the culprits behind the optic nerve degeneration in glaucoma [321]. Yang et al. have found that ferroptosis may not only be associated with RGC and optic nerve degeneration in glaucoma models but may also affect axon survival. Therefore, its contribution to RGC damage and optic nerve degeneration should not be overlooked [322]. Pathological high intraocular pressure (ph-IOP) is a critical feature in the development of glaucoma and a major factor leading to RGC loss [323]. Lowering ph-IOP through medical or surgical interventions is currently the only effective treatment for glaucoma in clinical practice. A recent study has reported the interplay between ferroptosis and glaucoma, unveiling a novel mechanism where ph-IOP induces retinal iron metabolism disruption and promotes RGC ferroptosis. They elucidated the functional characteristics of ferroptosis in glaucoma and emphasized how ph-IOP can disrupt iron homeostasis, leading to significant accumulation of free iron in the retina post-injury [324]. This iron accumulation disrupts the intracellular redox system, triggering RGC ferroptosis. NCOA4-mediated degradation of FTH1 is one of the reasons for intracellular iron accumulation post ph-IOP; knocking out NCOA4 inhibits FTH1 degradation, thereby alleviating iron accumulation. Oral administration of DFP can penetrate the blood-retinal barrier, effectively chelating excess free iron in the retina and ultimately preventing RGC ferroptosis. Additionally, DFP treatment provides significant morphological and functional protection for RGCs. These findings indicate that ferroptosis is a novel therapeutic target for treating glaucoma, especially in addressing the ongoing RGC loss following ph-IOP control [324].

In summary, ferroptosis, as a newly discovered mode of cell death, is a hot topic in current research, and numerous studies have demonstrated its close association with IBD. This section summarizes the potential signaling pathways involved in ferroptosis regulation in IBD. By exploring the mechanisms and relevant targets of ferroptosis, the occurrence of cellular ferroptosis can be regulated, effectively alleviating the progression of experimental animal IBD to a certain extent. However, there are still many questions waiting to be answered: What are the specific mechanisms of endogenous metabolic pathways in ferroptosis in IBD? How to control the dosage of iron intake? ROS is widely present in many cells, so how can intervention be conducted specifically? Besides intestinal epithelial cells, do intestinal immune cells also experience ferroptosis? Currently, drug therapy for IBD primarily focuses on immunosuppression, which cannot completely resolve the occurrence of intestinal inflammation. Therefore, further in-depth research elucidating the specific mechanisms and regulatory factors of ferroptosis is expected to provide new insights into potential therapeutic targets for IBD.

## Treatment and mechanism research on autoimmune diseases–inhibitors and inducers of ferroptosis

### Ferroptosis-related inducers

Precision drug interventions can be accomplished in the future by targeting pathogenic cells in autoimmune diseases, such as plasmacytoid dendritic cells involved in innate immune responses, inflammatory macrophages, and other cells responsible for secreting autoantibodies in the adaptive immune response pathways, as well as pathogenic cells in target organs such as synovial cells and fibroblasts. Achieving this precision drug intervention could involve employing ferroptosis inducers. Current research findings indicate that any substance, such as enzymes or proteins, capable of activating or deactivating key genes in the ferroptosis regulatory pathway (including genes related to iron metabolism, glutathione transport system, or regulation of lipid peroxidation) can play a role in regulating cell ferroptosis (Table 1). For instance, Erastin, a small molecule anticancer drug, functions by directly inhibiting System Xc and disrupting cellular antioxidant processes, thereby inducing tumor cell death. It was the first identified inducer of ferroptosis. Conversely, deferoxamine (DFO), an iron chelator, was the earliest compound discovered to inhibit erastin-induced cell ferroptosis. Several inducers and inhibitors of ferroptosis have been identified, primarily acting through the following mechanisms: 1) inhibition of System Xc-; 2) inhibition of GPX4/GSH; 3) induction of iron production; and 4) induction of cellular lipid peroxidation (Table 1).

**Table 1:** Ferroptosis-related inducers.

Mechanisms	Ferroptosis-related inducers	Functions	Reference.
Inhibit GPX4/GSH	Ras-selective Lethal Small Molecule 3 (RSL3)	Inhibit GPX4	[341]
	DMOCPTL	GPX4 ubiquitination	[331]
	FINO2	Indirectly inhibits GPX4, directly oxidizes iron	[332]
	L-buthionine (S,R)-Sulfoximine (BSO)	Reduce glutathione-dependent peroxidase activity	[333]
	Legumain	Inhibit GPX4	[334]
	APR-246	Deactivate SLC7A11 or GPX4	[334]
	FIN56	Promote GPX4 degradation	[335]
	Artesunate (ART)	Inhibit GPX4	[336]
	ML210/ ML162/JKE-1674/	Inhibit GPX4	[337]
Increase intracellular iron and induce lipid peroxidation	Lipopolysaccharide (LPS)	Inhibits GPX4 and induces ferroautophagy	[338]
	High-glucose	Increase MDA levels, ROS levels and intracellular iron ion levels	[339]
	EF24	Increase MDA levels, ROS levels and intracellular iron ion levels	[340]
	Angiotensin II (Ang II)	Increase ROS levels and inhibit GSH levels	[341]
	Oxidized-low density lipoprotein (ox-LDL)	Increase ROS, intracellular iron ion levels, and induce lipid peroxidation	[342]

### Ferroptosis-related inhibitors

**Fig. 10:**
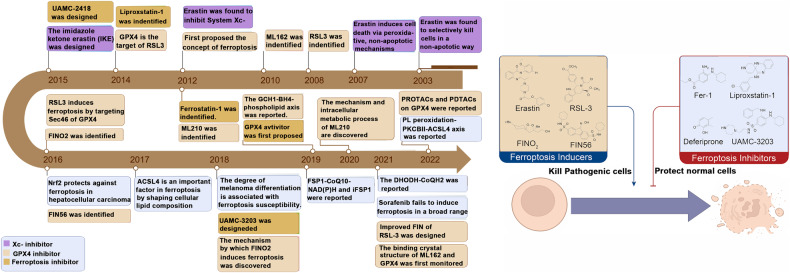
The key time points for the discovery of inhibitors and inducers of ferroptosis. Since 2003, many inhibitors and inducers of ferroptosis have been discovered. ACSL4 acyl-CoA synthetase long-chain family member 4, CoQ10 coenzyme Q10, GPX4 glutathione peroxidase 4.

In the future, precise drug interventions targeting normal functioning cells within target organ tissues in autoimmune diseases, such as podocytes in lupus nephritis, can be achieved using ferroptosis inhibitors. Current research has revealed that ferroptosis inhibitors primarily work by reducing intracellular free iron levels or enhancing cellular antioxidant capacity to improve cell ferroptosis (Table 2). Further research is necessary to delve into the specific regulatory mechanisms of ferroptosis inhibitors and inducers on cell ferroptosis, as well as their effects on autoimmune diseases. The key time points for the discovery of inhibitors and inducers of ferroptosis is shown in Fig. 10.

**Table 2:** Ferroptosis-related inhibitors.

Mechanisms	Ferroptosis-related inhibitors	Functions	Reference.
Iron chelators	Deferoxamine (DFO)	Reacts with Fe3+ to induce GPX4, xCT and glutathione expression	[343, 344]
	Deferasirox (DFX)	Binds Fe3+ to inhibit ROS generation	[345, 346]
	Ciclopirox	Binding Fe3+	[330]
	Deferiprone	Reduce iron levels, improve mitochondrial function, and reduce reactive oxygen species levels	[347]
	BMS536924	bound iron ions	[348]
Lipid peroxidation inhibitor or glutathione transport system activator	Liproxstatin-1 (Lip-1)	Inhibit mitochondrial lipid peroxidation and restore the expression of GSH, GPX4 and ferroptosis inhibitory protein 1	[349]
	ferrostatin-1 (Fer-1)	Induces GPX4 protein expression and reduces lipid ROS	[350]
	Quercetin (QCT)	Reduce MDA and lipid ROS levels and increase GSH levels	[351]
	Lapatinib	Restoring GPX4 expression	[352]
	SREBF2	Induces transferrin (TF) transcription and reduces intracellular iron pools, reactive oxygen species, and lipid peroxidation levels	[352]
	SRS 16-86	Upregulate the expression of GPX4, GSH and xCT and inhibit lipid peroxidation	[353]
	N-acetylcysteine (NAC)	Induces GPX4 expression	[354]
	Zileuton	Reduce ROS levels, lipoxygenase inhibitors	[355]
	CMS121	Reduce inflammation and lipid peroxidation	[356]
	Vitamin E	Reduce lipid peroxidation	[357]

## Prospects

In recent years, numerous studies have commenced exploring the interplay between ferroptosis, immune cells, and autoimmune diseases, accentuating the dual role of ferroptosis in pathology. Nevertheless, several queries necessitate further investigation. Primarily, unraveling the precise mechanisms governing iron-dependent cell demise in specific immune cells lacking Gpx4 remains elusive. Delving into the energy metabolism profiles of diverse immune cell types and the Gpx4-independent antioxidant pathways assumes importance in guiding the therapeutic strategies for associated conditions [325]. Secondly, why does cellular immunogenicity peak during early stages of cell ferroptosis, whereas both early and late stages of cell demise like apoptosis and necrosis exhibit robust immunogenicity [326, 327]? The diminishing immunogenicity observed in the later phase of ferroptosis poses a persistent enigma. It challenges the established notion of heightened immunogenic potential post late-stage cell demise, traditionally attributed to a surge in inflammatory mediators upon cell rupture. Unraveling the molecular underpinnings dictating the immunogenicity of cell ferroptosis warrants further elucidation. Thirdly, Macrophages secrete factors such as TGF-β1, while CD8 + T cells release IFNγ, influencing cell ferroptosis. Moreover, an array of immune factors can engage in the realm of ferroptosis research. Furthermore, evaluating the susceptibility of various bacterial strains to ferroptosis stands as an engaging pursuit. Fourthly, dissecting the mechanisms through which neutrophil extracellular traps (NETs) induce cell ferroptosis necessitates deeper scrutiny. NETs, characterized by fibrous structures measuring 15–17 nanometers and harboring spherical elements like histones H2, H3, and H4, elastase, myeloperoxidase, lactoferrin, and gelatinase [328], warrants attention. Although myeloperoxidase (MPO) in neutrophils can instigate cell ferroptosis, confirming whether MPO serves as the primary entity enabling METs to induce cell ferroptosis remains pending. Additionally, exploring the route through which MPO penetrates the parenchymal functional cells of target organs in autoimmune disorders via the cell membrane necessitates in-depth exploration. Fifthly, elucidating how ferroptosis impacts autoimmune diseases may unveil prospective targets for future therapeutics or diagnostics. For instance, the development of inhibitors targeting DAMPs and pertinent nanomaterial-based drugs could prove instrumental [328].

Moreover, the involvement of ferroptosis in autoimmune disorders is still a subject of ongoing investigation, marked by numerous outstanding queries. Primarily, the precise mechanisms governing ferroptosis remain enigmatic. Despite indications from various studies suggesting that ferroptosis assessment can hinge on iron levels, ROS, GPX4 expression, and cell viability, the absence of a definitive biomarker specific to ferroptosis looms large [329]. It becomes imperative for upcoming research to pinpoint distinct markers exclusive to ferroptosis to comprehensively interrogate this process. While aberrations in iron metabolism and lipid peroxidation may pave the path for ferroptotic processes under pathological states, the underlying intricacies necessitate deeper exploration. Notably, the modulation of iron metabolism regulatory elements like TfR1, DMT1, and transferrin protein 1 can potentially sway the onset and intensity of ferroptosis. Yet, the query persists regarding whether these proteins possess the capacity to impact alternative signal cascades and if metals beyond iron can instigate ferroptosis through distinct molecular pathways. Recent investigations have unearthed a novel player in the ferroptosis domain, MBOAT2, functioning autonomously of GPX4 and FSP1, overseeing ferroptosis surveillance through phospholipid remodeling and proficiently impeding ferroptotic processes. Consequently, forthcoming explorations should unravel other autonomous routes instrumental in mediating ferroptosis. Furthermore, specific iron chelators (such as deferoxamine, deferiprone), chloroquine and its derivatives, antioxidants (e.g., vitamin E, alpha-lipoic acid, selenium), chloroquine analogs like Fer-1, and calreticulin have showcased therapeutic promise in preclinical trials for autoimmune ailments. Nevertheless, the efficacy of these interventions necessitates validation through multicenter randomized controlled clinical trials. Subsequent investigations will pivot towards the feasibility of manipulating factors linked with ferroptosis, proffering pivotal pathways for the scrutiny and advancement of treatments geared towards autoimmune diseases.

**Fig. 11:**
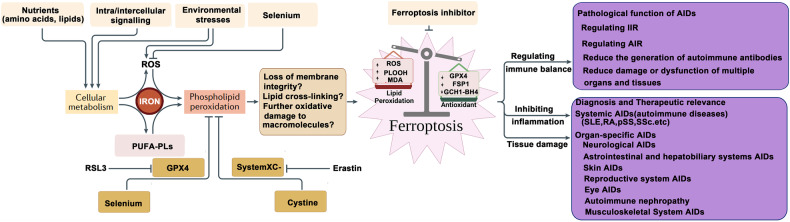
Prospects on ferroptosis in various autoimmune diseases. Summary of the potential mechanisms of ferroptosis in various autoimmune diseases. GPX4 glutathione peroxidase 4, ROS reactive oxygen species, AD autoimmune diseases.

**Fig. 12:**
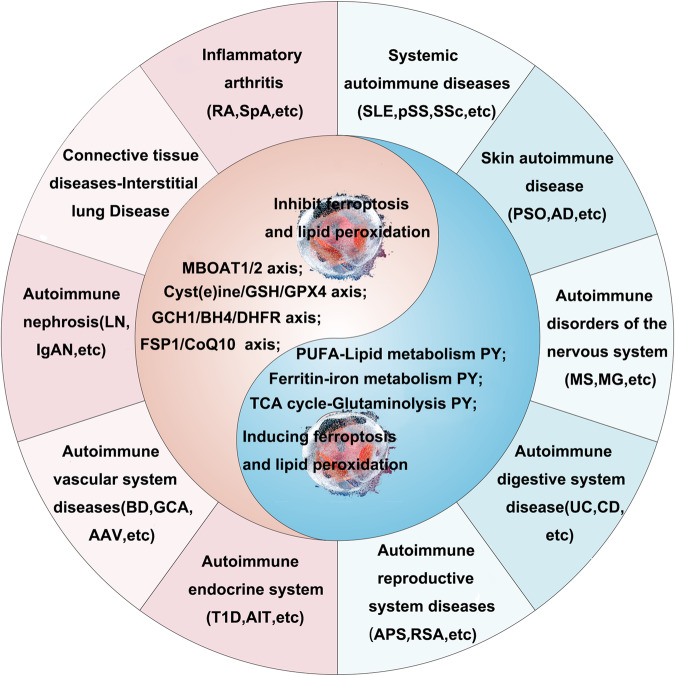
Ferroptosis and autoimmune diseases. Ferroptosis is involved in a variety of autoimmune diseases. PUFA polyunsaturated fatty acid, GPX4 glutathione peroxidase 4, GSH glutathione, FSP1 ferroptosis inhibitor protein 1.

In summary, research on the role of ferroptosis in autoimmune diseases and its underlying molecular mechanisms is still in its early stages. Current data mainly come from cell and animal models, highlighting the need for clinical studies in autoimmune disease patients. The development of drugs targeting ferroptosis in autoimmune diseases is an important research direction. In-depth understanding of the mechanisms of ferroptosis provides molecular targets for suppressing inflammation and excessive immune responses in autoimmune diseases. However, during the onset and progression of autoimmune diseases, immune cells and parenchymal cells in target organs may undergo various types of programmed cell death, including pyroptosis, autophagy, necrosis, and apoptosis. Key questions include which types of programmed cell death to focus on, whether combination therapies targeting multiple forms of programmed cell death are necessary, and when to intervene. These issues require further basic and clinical research to be resolved (Fig. 11 and Fig. 12).

## Data Availability

All data generated or analyzed during this study are included in this published article.
