# Author Correction: Advances in research on immunocyte iron metabolism, ferroptosis, and their regulatory roles in autoimmune and autoinflammatory diseases

**DOI:** 10.1038/s41419-024-07112-8

**Published:** 2024-10-21

**Authors:** Liuting Zeng, Kailin Yang, Ganpeng Yu, Wensa Hao, Xiaofei Zhu, Anqi Ge, Junpeng Chen, Lingyun Sun

**Affiliations:** 1 Department of Rheumatology and Immunology, Nanjing Drum Tower Hospital, Chinese Academy of Medical Sciences and Peking Union Medical College, Graduate School of Peking Union Medical College, Nanjing, China; 2 Key Laboratory of Hunan Province for Integrated Traditional Chinese and Western Medicine on Prevention and Treatment of Cardio-Cerebral Diseases, School of Integrated Chinese and Western Medicine, Hunan University of Chinese Medicine, Changsha, China; 3 Psychosomatic laboratory, Department of Psychiatry, Daqing Hospital of Traditional Chinese Medicine, Daqing, China; 4 People’s Hospital of Ningxiang City, Ningxiang, China; 5 Institute of Materia Medica, Chinese Academy of Medical Sciences & Peking Union Medical College, Beijing, China; 6 Fudan University, Shanghai, China; 7 The First Hospital of Hunan University of Chinese Medicine, Changsha, Hunan China; 8 Department of Physiology, School of Medicine, University of Louisville, Louisville, KY USA; 9 College of Mechanical Engineering, Hunan University of Science and Technology, Xiangtan, China; 10 Department of Rheumatology and Immunology, The First Affiliated Hospital of Anhui Medical University, Hefei, China

**Keywords:** Immunology, Immunological disorders

Correction to: *Cell Death and Disease* 10.1038/s41419-024-06807-2, published online 04 July 2024

In this article the figure captions have been published without the image using license. This has been corrected:

Fig. 2 The molecular mechanism of ferroptosis {figures adapted from Zhang et al. 2023 [358], licensed under CC BY 4.0}.

Fig. 3 Fatty acid biosynthesis and ferroptosis {figures adapted from Kim et al. 2023 [359], licensed under CC BY 4.0}.

Fig. 6 The pathways affecting the metabolic basis of ferroptosis {figures cited from Yuan et al. 2024 [360], licensed under CC BY-NC-ND 4.0}.

Reference list:

[358] Zhang T, Luo L, He Q, et al. Research advances on molecular mechanism and natural product therapy of iron metabolism in heart failure. Eur J Med Res. 2024;29:253. 10.1186/s40001-024-01809-4

[359] Kim JW, Lee JY, Oh M, et al. An integrated view of lipid metabolism in ferroptosis revisited via lipidomic analysis. Exp Mol Med. 2023;55:1620–31. 10.1038/s12276-023-01077-y

[360] Yuan M, He Q, Xiang W, Deng Y, Lin S, Zhang R. Natural compounds efficacy in ophthalmic diseases: a new twist impacting ferroptosis. Biomed Pharmacother. 2024;172:116230. 10.1016/j.biopha.2024.116230

The original article has been corrected.

